# Sex-Specific Effects of Synbiotic Exposure in Mice on Addictive-Like Behavioral Alterations Induced by Chronic Alcohol Intake Are Associated With Changes in Specific Gut Bacterial Taxa and Brain Tryptophan Metabolism

**DOI:** 10.3389/fnut.2021.750333

**Published:** 2021-11-26

**Authors:** Nieves Pizarro, Elk Kossatz, Pedro González, Alba Gamero, Emma Veza, Cristina Fernández, Toni Gabaldón, Rafael de la Torre, Patricia Robledo

**Affiliations:** ^1^Integrative Pharmacology and Systems Neuroscience Research Group, Neurosciences Research Program, IMIM-Hospital del Mar Medical Research Institute, Barcelona, Spain; ^2^Department of Experimental and Health Sciences, Pompeu Fabra University (CEXS-UPF), Barcelona, Spain; ^3^Microomics Systems S.L., Barcelona, Spain; ^4^Barcelona Supercomputing Centre (BSC-CNS), Barcelona, Spain; ^5^Institute for Research in Biomedicine, The Barcelona Institute of Science and Technology, Barcelona, Spain; ^6^Catalan Institution for Research and Advanced Studies (ICREA), Barcelona, Spain; ^7^CIBER Fisiopatología Obesidad y Nutrición, Instituto de Salud Carlos III, Madrid, Spain

**Keywords:** synbiotic, microbiota, alcohol, addiction, serotonin, prefrontal cortex, hippocampus, sex differences

## Abstract

Chronic alcohol intake has been shown to disrupt gut microbiota homeostasis, but whether microbiota modulation could prevent behavioral alterations associated with chronic alcohol intake remains unknown. We investigated the effects of synbiotic dietary supplementation on the development of alcohol-related addictive behavior in female and male mice and evaluated whether these effects were associated with changes in bacterial species abundance, short-chain fatty acids, tryptophan metabolism, and neurotransmitter levels in the prefrontal cortex and hippocampus. Chronic intermittent exposure to alcohol during 20 days induced escalation of intake in both female and male mice. Following alcohol deprivation, relapse-like behavior was observed in both sexes, but anxiogenic and cognitive deficits were present only in females. Synbiotic treatment reduced escalation and relapse to alcohol intake in females and males. In addition, the anxiogenic-like state and cognitive deficits observed in females following alcohol deprivation were abolished in mice exposed to synbiotic. Alcohol-induced differential alterations in microbial diversity and abundance in both sexes. In females, synbiotic exposure abrogated the alterations provoked by alcohol in *Prevotellaceae* UCG-001 and *Ruminococcaceae* UCG-014 abundance. In males, synbiotic exposure restored the changes induced by alcohol in *Akkermansia* and *Muribaculum* uncultured bacterium abundance. Following alcohol withdrawal, tryptophan metabolites, noradrenaline, dopamine, and γ-aminobutyric acid concentrations in the prefrontal cortex and the hippocampus were correlated with bacterial abundance and behavioral alterations in a sex-dependent manner. These results suggested that a dietary intervention with a synbiotic to reduce gut dysbiosis during chronic alcohol intake may impact differently the gut-brain-axis in females and males.

## Introduction

According to the WHO (1), alcohol misuse causes ~3 million deaths every year, and the public health burden of associated mental and behavioral disorders is very high (2). Excessive alcohol consumption induces addiction and dependence related to maladaptive changes in brain neurotransmitters that can persist even after protracted abstinence, which cause cravings and relapse when stimuli associated with alcohol consumption appear (3). Although clear sex differences have been observed in alcohol use disorders, with males drinking more than females; recent data indicate that this gap is being reduced, especially in young people, where alcohol drinking patterns are changing [see (4) for review]. For instance, the prevalence of frequent binges in younger women is increasing to resemble those of men (4, 5). In general, these data underscore the relevance of including both sexes in studies aimed at investigating the biological factors contributing to the development of alcohol use disorders, and to finding new treatment avenues to prevent the alcohol-related addictive process and the associated neuropathological consequences.

Microbiota is the population of microorganisms (mainly bacteria), which live symbiotically in our body. The composition of the intestinal microbiota is established in the 1st years of life, although there are fluctuations in its composition depending on lifestyle and diet. There is evidence that gut microbiota regulates many aspects of its host's physiology through modulation of the immune system (6), and increasing evidence points to the influence of gut microbiota on the central nervous system (CNS) through the gut-brain axis (7, 8). Thus, it has been shown that emotional and cognitive alterations are closely linked to dysbiosis (9–11). Short-chain fatty acids (SCFA) are the main metabolites produced by bacterial fermentation of dietary fiber, which possesses powerful anti-inflammatory properties. In particular, several species of bacteria including *Bifidobacterium* and *Lactobacillus* have been shown to produce acetate, butyrate, isobutyrate, and propionate that have neuroactive properties (12). In addition, some of these bacteria can produce neurotransmitters such as γ-aminobutyric acid (GABA), serotonin (5-HT), dopamine (DA), and noradrenaline (NA) (13), which play key roles in the pathophysiology of several CNS diseases, including anxiety, depression, and addiction (14, 15). Moreover, gut microbes metabolize their energy resources *via* the tryptophan (Tryp) pathway, which regulates the availability of circulating 5-HT and kynurenine (Kyn), known neuroactive metabolites involved in the pathophysiology of several CNS diseases (16, 17).

Alcohol use produces intestinal bacterial overgrowth and dysbiosis that can lead to intestinal hyperpermeability or leaky gut and reduced production of antimicrobial peptides (18). An increase in gut epithelium permeability is usually followed by endotoxins releases, such as lipopolysaccharides and peptidoglycans that conglomerate in the portal circulation, enhancing the response of B cells and the key transcription factor NF-KB. This is accompanied by pro-inflammatory cytokines release, with the consequent increment of reactive oxygen species and hence oxidative stress, causing among others, hepatocellular damage [reviewed in (16)]. In addition, it has been shown that chronic alcohol use decreases beneficial gut bacteria, such as *Bifidobacterium*, and increases potentially damaging bacteria, such as the obesity-linked *Proteobacteria* (19–21). Importantly, alterations in gut microbiota caused by alcohol exposure have been associated with neuropathological disorders such as depression and anxiety in humans and animal models (22–25). However, there is still a lack of data evaluating whether microbiota alterations induced by chronic alcohol intake could contribute to the development of alcohol addiction and whether sex differences exist in this interaction.

The restoration of dysbiosis in microbiota has been approached from many perspectives. Recently, modulation of intestinal microbiota with the use of dietary supplements such as pre- or probiotics has been suggested as a novel therapeutic target in the treatment of cognitive and behavioral pathologies (22, 26–28). Studies show that *Lactobacillus* and *Bifidobacterium* are capable of producing GABA from glutamate in culture (29), and that *Lactobacillus rhamnosus* is capable of modulating the central expression of GABAergic receptors in brain regions of the CNS (30). The administration of *Bifidobacterium infantis* regulates DA and 5-HT metabolism in the frontal cortex, increase plasma concentrations of Tryp, and reduce depressive-like behaviors in rats (9). All these findings suggest that certain prebiotics, probiotics, or their combination (i.e., synbiotic supplementation), may have the ability to modulate brain neurotransmitters and could attenuate alcohol-related addictive processes, and the associated affective and cognitive-behavioral disturbances.

Thus, the aim of this study was to investigate the role of gut microbiota on the development of alcohol-related addictive behaviors in female and male mice, through the modulation of gut bacterial species with dietary supplementation of synbiotic, and associate these alcohol-related effects with changes in gut bacterial species abundance, changes in fecal SCFA, in Tryp metabolism, DA, NA, and GABA concentrations in the prefrontal cortex (PFC) and hippocampus (HPC).

## Materials and Methods

### Animals and Behavioral Procedures

Adult C57BL/6J (7–9 weeks) female (*n* = 22) and male mice (*n* = 26) were housed in a room with controlled temperature (21 ± 1°C) and humidity (50 ± 10%), with an inverted 12 h light/dark cycle (lights off at 8:00 am; on at 8:00 pm), and standard chow available *ad libitum*. In order to guarantee that mice would consume relevant quantities of the synbiotic supplementation throughout the entire procedure and that alcohol would not affect synbiotic intake, we first carried out a pilot study evaluating the pattern and amount of alcohol (15% ethanol solution in water), water, and synbiotic (see below for composition) intake in male mice. Two groups of mice were presented with the choice between two drinking bottles for 21 days. On odd days (1, 3, 5, 7, 9, 11, 13, 15, 17, 19, and 21), mice had access to one bottle containing alcohol (15% ethanol in water), and another bottle containing water (group 1) or synbiotic (group 2). On even days (2, 4, 6, 8, 10, 12, 14, 16, 18, and 20), mice had access to two bottles of water (group 1) or synbiotic (group 2). The quantities of alcohol, water, and synbiotic consumed in 24 h were measured daily. The results are presented in Supplementary Figure 1. No significant differences were observed between groups in the amount of water or synbiotic consumed on even days, and the amount of synbiotic consumed did not vary during the procedure.

In the experimental procedure, both female and male mice were used. One group of females (*n* = 8) and males (*n* = 10) had free access to two drinking bottles in their home cages containing a mixture of the following probiotics (*Lactobacillus rhamnosus, Lactobacillus acidophilus, Lactobacillus casei, Lactobacillus plantarum, Lactobacillus salivarius, Lactobacillus paracasei, Bifidobacterium bifidum, Bifidobacterium lactis, Bifidobacterium infantis, Bifidobacterium longum*, and *Bifidobacterium breve*), and the prebiotic inulin for 2 weeks. The mixture (Now Foods Probiotic-10, Bloomingdale, IL, USA) containing 1 × 10^9^ colony-forming units, was prepared fresh every day dissolved in drinking water. Another group of females (*n* = 8) and males (*n* = 10) had access to two bottles containing only water for 2 weeks. Fecal samples were collected after this basal procedure. Subsequently, these groups of mice had intermittent voluntary access to alcohol [15% ethanol (ETOH) solution in water] in 24 h-cycles, on alternate days for 20 days following a modified version of the previously described intermittent alcohol access model (31). On odd days (1, 3, 5, 7, 9, 11, 13, 15, 17, and 19), mice were presented with the choice between two drinking bottles beginning 4 h after the start of the dark cycle. In one group of mice, one bottle contained alcohol 15%, and another bottle contained water (Group 1: water group). In another group of mice, one bottle contained alcohol 15%, and the other contained synbiotic (Group 2: synbiotic group). The position of the bottles was alternated to avoid the potential confounding effects of place preference. On even days (2, 4, 6, 8, 10, 12, 14, 16, 18, and 20), Group 1 had access to two bottles of water, and Group 2 had access to two bottles of synbiotic. In addition, separate groups of females (*n* = 6) and males (*n* = 6) only received water during the entire paradigm and served as controls.

**Figure 1:**
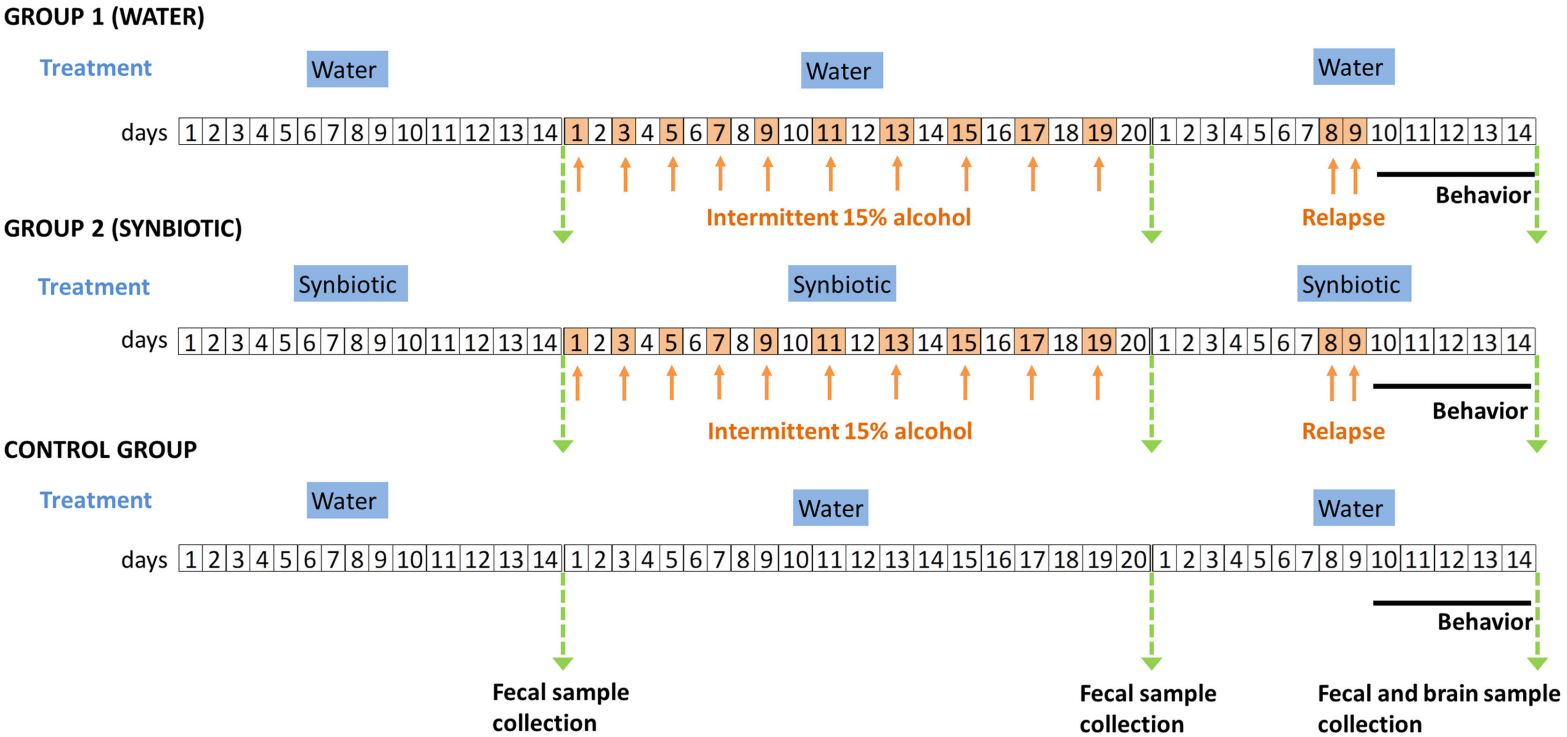
Schematic diagram of the experimental procedures followed in the study. Group 1 (males and females) were treated with water and Group 2 (males and females) were treated with synbiotic during the entire procedure. The control group was treated with water and never received alcohol, and was included in the post-relapse behavioral analyses. The timeline of the experiments (days) is shown in squares. White squares represent days where mice drank water plus the corresponding treatment (in blue titles), and orange squares (and arrows) represent the days where mice drank alcohol (15% ethanol solution in water) plus the corresponding treatment. Green arrows represent the days of sample collection. The solid black line indicates the days of post-relapse behavioral testing.

Subsequently, alcohol was removed for 7 days, but each group continued to have access to drinking bottles containing either water or synbiotic. The next day, mice were tested in the alcohol deprivation effect model, similar to the one previously described (31), where mice had again 24 h access to one bottle with the 15% alcohol solution, and another bottle with either water or synbiotic. Mice were again deprived of alcohol for 2 days, and behavioral testing was performed during withdrawal. The marble-burying (MB), the novel object recognition (NOR), and the tail suspension (TS) tests were performed. All tests were conducted by an observer blind to the experimental conditions. The MB test was performed first to evaluate the anxiety-related behavior. Mice were placed for 20 min in a plastic box (30 cm wide × 55 cm long) filled with 5 cm depth of wood chip bedding with 12 marbles uniformly spaced. After 20 min, the number of marbles buried (2/3 of the marble-covered with bedding) was measured. Secondly, the NOR test was performed to evaluate long-term memory deficits. For this test, a black Plexiglas maze with two corridors (30 cm long × 4.5 cm wide × 15 cm high walls) set at a 90° angle (Panlab, Spain) was used. This test consists of three phases, one each 24 h (habituation, training, and testing). On the 1st day, mice were habituated to the empty maze for 9 min. On the 2nd day, mice were trained in the maze for 9 min, but this time two identical objects were presented, one on each arm of the maze. On the 3rd day, mice were tested in the maze for 9 min, but one of the familiar objects was replaced by a novel object, and the total time spent exploring each object (novel and familiar) was recorded. A discrimination index was calculated as the time spent exploring the novel object minus the time exploring the familiar object divided by the total time exploring both objects. A higher discrimination index reflects greater memory retention. Lastly, the TS test was performed to evaluate depression-related behaviors. In a bar, 30 cm above the floor, mice were suspended by their tails with tape for 6 min. In the last 4 min, the time that mice spent as immobile was measured. Fresh fecal samples from mice were collected after the basal procedure and the end of the study weighed and stored immediately at −80°C until analysis. Finally, mice were euthanized after behavioral testing, and brain areas were harvested, weighed, and stored immediately at −80°C until analysis. A schematic diagram of the entire procedure followed in the study is shown in Figure 1.

### Library Preparation and Sequencing

Deoxyribonucleic acid was extracted from 36 samples using the MagMAX CORE Nucleic Acid Purification Kit 500RXN (Thermo Fisher, Austin, USA), following the instructions of the manufacturer. Mock community DNA was included as a positive control for library preparation (Zymobiomics Microbial Community DNA, ZymoResearch, Irvine, CA, USA). Samples were amplified using 16S rRNA V3-V4 regions specific primers (V3-V4-Forward 5′-TCGTCGGCAGCGTCAGATGTGTATAAGAGACAGCCTAC GGGNGGCWGCAG-3′, V3-V4-Reverse 5′GTCTCGTGGGCT CGGAGATGTGTATAAGAGACAGGACTACHVGGGTATCT AATCC-3′).

The PCR was performed in 10 μl final volume with 0.2 μM primer concentration. The PCR cycle included: 3 min at 95°C (initial denaturation) followed by 25 cycles: 30 s at 95°C 30 s at 55°C, and 30 s at 72°C, and a final elongation step of 5 min at 72°C. PCR products were purified using AMPure XP beads (Beckman Coulter, Nyon, Switzerland) with a 0.9 × ratio according to the instructions of the manufacturer. The above-described primers contain overhangs allowing the addition of full-length Nextera barcoded adapters for multiplex sequencing in a second PCR step, resulting in sequencing-ready libraries with ~450 bp insert sizes. In brief, 5 μl of the first PCR purified product were used as a template for a second PCR with Nextera XT v2 adaptor primers in a final volume of 30 μl using the same PCR mix and thermal profile as for the first PCR but with only eight cycles. Afterward, 25 μl of the second PCR product were purified with SequalPrep normalization kit (Invitrogen, ThermoFisher Scientific, Waltham, MA, USA), according to the protocol of the manufacturer. Libraries were eluted in 20 μl final volume and pooled for sequencing. The final pool was quantified by qPCR using the Kapa library quantification kit for Illumina Platforms (Kapa Biosystems, SigmaAldrich, Saint Louis, MO, USA) on an ABI 7900HT real-time cycler (Applied Biosystems, ThermoFisher Scientific, Waltham, MA, USA). Sequencing was performed using Illumina MiSeq (San Diego, USA) (2 × 300 bp) and v3 chemistry with a loading concentration of 10 pM. In all cases, 15% of PhIX control libraries were used to increase the diversity of the sequenced sample. Negative controls included sample collection buffer, DNA extraction, and PCR amplification steps, PRC products after both PCR steps were visualized by electrophoresis gel (1.5% agarose) with SYBR Safe (ThermoFisher Scientific, Waltham, MA, USA). No visible bands were observed.

### Amplicon Sequences Processing and Analysis

Raw demultiplexed forward and reverse reads were processed using the following methods and pipelines as implemented in QIIME2 version 2019.4 with default parameters unless stated (32). DADA2 was used for quality filtering, denoising, pair-end merging, and amplicon sequence variant calling (ASV; i.e., phylotypes) using the qiime dada2 denoise-paired method (33). Q20 was used as a quality threshold to define read sizes for trimming before merging (parameters: –p-trunc-len-f and –p-trunc-len-r). Reads were truncated at the position when the 75th percentile Phred score felt below Q20: 266 bp for forwarding reads and 224 bp for reverse reads. After quality filtering steps, the average sample size was 94.266 reads (min: 22.09 reads, max: 155.084 reads) and 4.144 phylotypes were detected. ASVs were aligned using the qiime alignment mafft method (34). The alignment was used to create a tree and to calculate phylogenetic relations between ASVs using the qiime phylogeny fast tree method (35). ASV tables were subsampled without replacement in order to even sample sizes for diversity analysis using qiime diversity core-metrics-phylogenetic pipeline. The smallest sample size was chosen for subsampling. Jaccard, Bray Curtis, and unweighted and weighted Unifrac distances (36) were calculated to compare community structure. Alpha diversity metrics calculated included observed operational taxonomic unit (OTU) number and ACE index (i.e., richness), and Simpson index (i.e., diversity). Taxonomic assignment of ASVs was performed using a Bayesian Classifier trained with Silva database (i.e., 99% OTUs database) using the qiime feature-classifier classify-sklearn method (37). Phylotypes were filtered to discard contaminant Eukariota DNA-derived amplicons using Blast against the mentioned database with a 90% identity cut-off. Only those microbial species that showed a percentage of relative abundance superior to 1% were considered for statistical analyses of the effects of alcohol and synbiotic exposure in female and male mice.

### Quantification of Fecal SCFA, PFC, and HPC Concentrations of Tryp Metabolites and Neurotransmitters

To quantify butyric and propionic acids concentrations, fecal samples were homogenized with milli-Q water/acetonitrile (1:1). Following centrifugation, the supernatant was diluted to achieve a concentration of 100 μg/ml. The analytical procedure was adapted for fecal samples from a high performance liquid chromatography tandem mass spectrometry (HPLC-MS/MS) methodology described by Gomez-Gomez et al. (38) for urine samples. Briefly, a derivatization procedure was implemented consisting in the formation of oxime ethers by the reaction of *o*-benzylhydroxylamine, and *N*-(3-dimethylaminopropyl)-*N*′-ethylcarbodiimide hydrochloride with the SCFA carboxylic acid chemical group. After a liquid-liquid extraction and evaporation of the organic phase, extracts were reconstituted and analyzed. The chromatographic and MS/MS conditions were optimized by using both mice and human fecal samples. Endogenous concentration levels for butyric and propionic acids were determined with the standard addition approach, as previously described (39). Quantification of Tryp metabolites (Tryp, 5-HT, 5-hydroxyindoleacetic acid: 5-HIAA, and Kyn) in PFC and HPC samples was performed according to a previously validated methodology (40). In addition, the concentrations of several neurotransmitters, including DA, NA, and GABA were quantified in PFC and HPC samples, as previously described (41). These analytical procedures were performed using an Acquity I Class UPLC system (Waters Associates, Milford, MA, USA) coupled to a triple quadrupole (TQS Micro) mass spectrometer provided with an orthogonal Z-spray-electrospray interface (ESI) (Waters Associates, Milford, MA, USA). The chromatographic separation was achieved using an Acquity BEH C18 column (100 × 2.1 mm i.d., 1.7 μm) (Waters Associates, Milford, MA, USA).

### Statistical Analysis

To evaluate the normality of the data distribution we applied the Kolmogorov-Smirnoff normality test. The escalation of alcohol intake and preference ratios were analyzed separately for females and males using a two-way ANOVA with treatment (water or synbiotic) as between-subject factors, and day (day 1 of alcohol intake and day 10 of alcohol intake) as a within-subjects factor. The relapse data were analyzed using a two-way ANOVA with treatment (water or synbiotic) and sex (females and males) as between-subject factors. The post-relapse behavioral results were analyzed with a two-way ANOVA with treatment (only water, water-alcohol, and synbiotic-alcohol) and sex (females and males) as between-subject factors. The PCF and HPC concentrations of Tryp metabolites, DA, NA, and GABA were analyzed separately for females and males with a one-way ANOVA comparing the water only, water-alcohol, and synbiotic-alcohol groups. The concentrations of SCFA in fecal samples, the alpha diversity data, and the relative abundances of bacterial taxa were analyzed separately in females and males with a two-way ANOVA with alcohol intake (no alcohol in basal conditions and alcohol intake on day 10) as a within-subjects factor, and treatment (water and synbiotic) as between-subjects factor. In all these cases, the LSD *post-host* test was applied as appropriate. Differences in basal bacterial abundance between females and males were analyzed with the Student's *t*-test. Pearson correlation analyses were carried out for females and males separately between behavioral parameters, bacterial species abundance, fecal SCFA, brain Tryp metabolites, and neurotransmitter concentrations. These statistical analyses were carried out using the GraphPad Prism software 8.0.1., (San Diego, USA) where significance was set a *p* < 0.05. Beta diversity distance matrices and ASV tables were used to calculate principal coordinates (PCoA) and construct ordination plots. The significance of groups in community structure was tested using Permanova. Permdisp test was used to identify location vs. dispersion effects (42). BiodiversityR version 2.11-1, PMCMR version 4.3, RVAideMemoire version 0.9–7 and vegan version 2.5–5 packages, and R software package version 3.6 (Viena, Austria) (http://www.R-project.org) were used.

## Results

### Effects of Synbiotic Intake on Escalation of Alcohol Consumption

**Figure 2:**
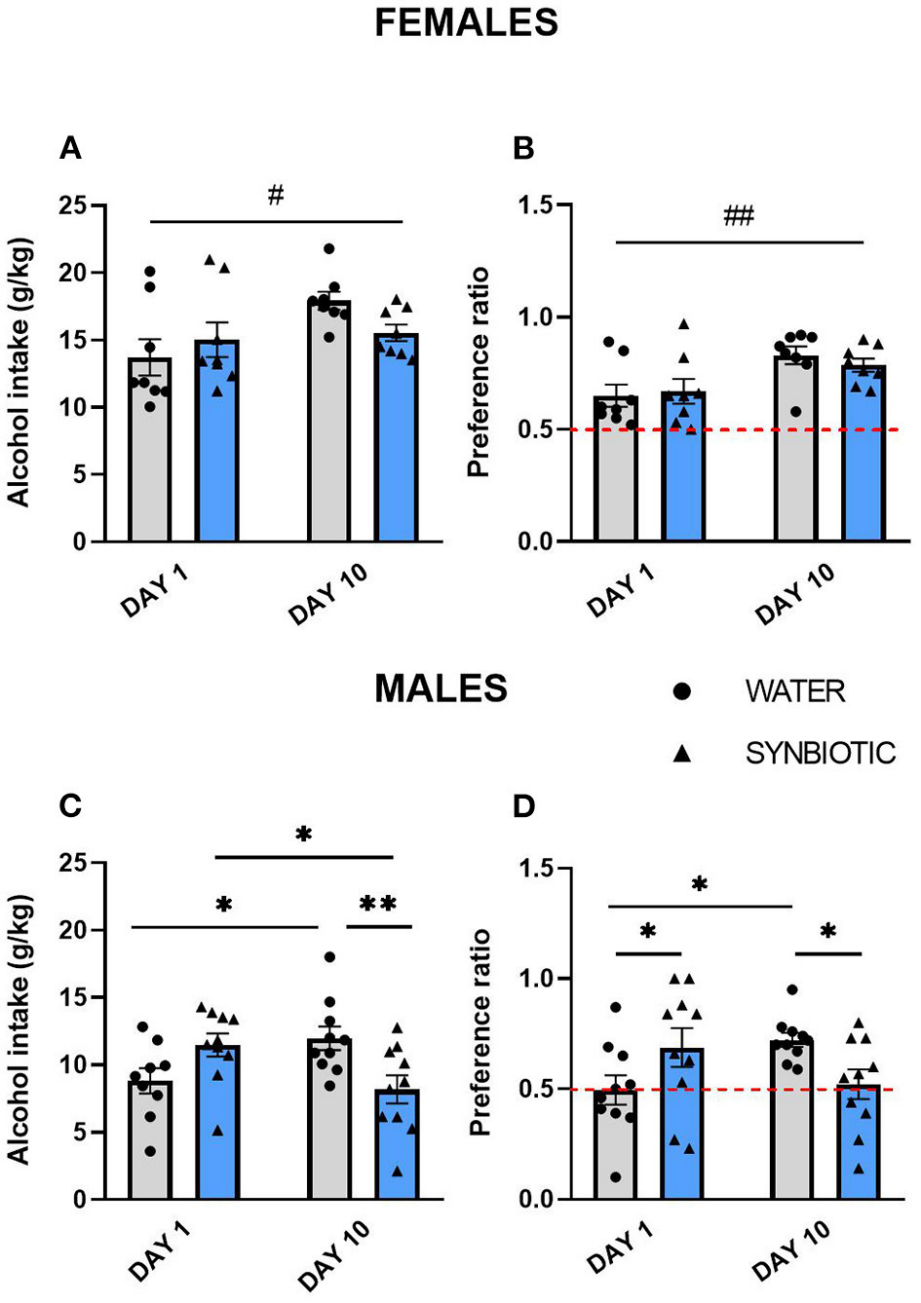
Alcohol intake and preference during intermittent access to alcohol (15% ethanol solution in water) in female and male mice treated with water (gray bars and round symbols), or synbiotic (blue bars, triangular symbols). **(A)** Female mice treated with water drank more alcohol (g/kg) during 24 h on day 10 with respect to day 1 (main effect of day, *p* < 0.05), while mice in the synbiotic group drank similar amounts of alcohol on both days. **(B)** Preference for alcohol in females increased on day 10 vs. 1 in both the water and the synbiotic groups (main effect of day, *p* < 0.01). **(C)** Male mice treated with water drank significantly more alcohol (g/kg) during 24 h on day 10 vs. day 1 (*p* < 0.05), while alcohol intake was significantly reduced on day 10 vs. day 1 in males treated with synbiotic (*p* < 0.05). In addition, on day 10 males drank less alcohol in the synbiotic vs. the water group (*p* < 0.01). **(D)** Preference for alcohol on day 10 was significantly increased vs. day 1 in the water group (*p* < 0.05), whereas the alcohol preference was reduced in the synbiotic group (*p* < 0.05). In addition, in males, synbiotic treatment increased preference for alcohol on day 1 with respect to water (*p* < 0.05). ^#^*p* < 0.05; ^##^*p* < 0.01 (main effect of day); *p < 0.05; **p < 0.01 (LSD *post-hoc* test following significant interaction in two-way ANOVA).

The data for intermittent alcohol intake in females and males are shown in Figure 2. In females, the water group showed an increase in alcohol consumption on the last day of exposure, while the synbiotic group did not show escalation to alcohol intake (Figure 2A) (significant main effect of the day: [*F*_(1, 28)_ = 5.15, *p* < 0.05]. Preference for alcohol in females was also increased from day 1 to day 10, but this effect was similar in both groups (Figure 2B) {significant main effect of the day: [*F*_(1, 28)_ = 11.07, *p* < 0.01]}. In males, the water group increased their alcohol intake from day 1 to 10, but not the synbiotic group (Figure 2C). Thus, a significant interaction was observed between treatment and day [*F*_(1, 35)_ = 11.96, *p* < 0.01]. *Post-hoc* comparisons showed that alcohol intake in males increased significantly from day 1 to 10 in the water group (*p* < 0.05), revealing an escalation to high alcohol intake. In contrast, males in the synbiotic group showed a significant decrease in alcohol intake on day 10 in comparison to day 1 (*p* < 0.05) (no escalation to high alcohol consumption), and a significant difference in alcohol intake was observed on day 10 between the water and synbiotic groups (*p* < 0.01). For alcohol preference in males, a significant interaction between treatment and day was observed [*F*_(1, 36)_ = 8.73, *p* < 0.01]. *Post-hoc* comparisons revealed a significant increase in preference from day 1 to 10 in the water group (*p* < 0.05), but not in the synbiotic group. On day 1 alcohol preference was significantly higher in the synbiotic group (*p* < 0.05), while on day 10, alcohol preference was significantly lower in the synbiotic group compared with the water group (*p* < 0.05; Figure 2D).

### Effects of Synbiotic Treatment on Alcohol Intake Following Alcohol Deprivation

**Figure 3:**
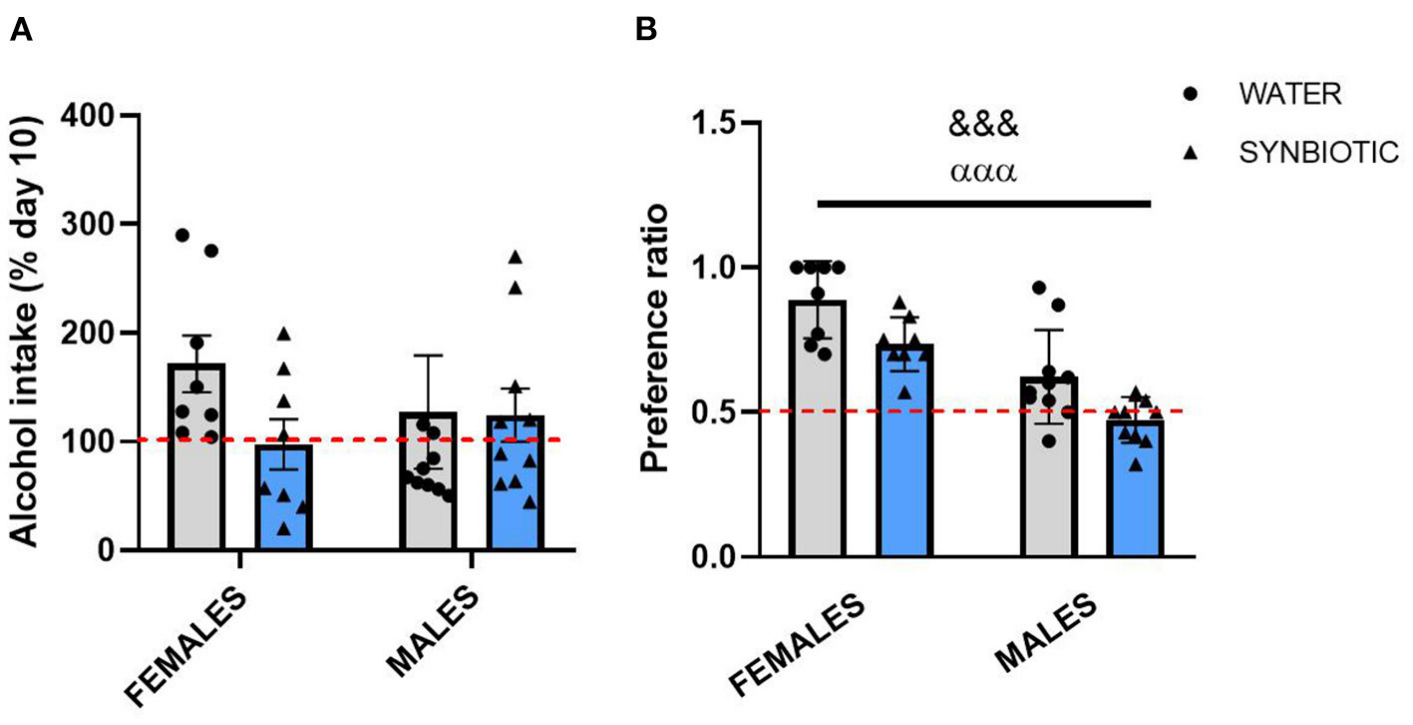
Alcohol intake and preference after alcohol deprivation in female and male mice treated with water (gray bars and round symbols), or synbiotic (blue bars and triangular symbols). Following repeated intermittent exposure to alcohol (15% ethanol solution in water), mice were deprived of alcohol for 7 days and subsequently tested in abstinence. Alcohol intake (% of day 10) and the alcohol preference ratio were quantified during the first 4 h of access. **(A)** For alcohol intake, no significant interaction or main effects were observed. However, females treated with water showed an increase in alcohol intake vs. day 10, but not those treated with synbiotic; and males treated with water also showed a slight increase in alcohol consumption, but this effect was similar in the synbiotic group. **(B)** Both females and males treated with water showed a preference for alcohol, but the preference ratio was significantly lower in males than in females. Both females and males treated with synbiotic showed less preference for alcohol than those treated with water. ^&&&^*p* < 0.01 (main effect of sex); ^ααα^*p* < 0.01 (main effect of treatment) two-way ANOVA.

Statistical analysis for alcohol intake (% of day 10) following 7 days of alcohol deprivation in female and male mice (Figure 3A) did not reveal a significant interaction between sex and treatment or any significant main effects. However, female mice in the water group showed an increase in alcohol consumption with respect to day 10, but not those mice treated with synbiotic. Males in the water group also consumed slightly more alcohol vs. day 10, but this effect was similar in the synbiotic group. The analysis of the preference ratio for alcohol in female and male mice (Figure 3B) revealed a main effect of sex [*F*_(1, 32)_ = 41.32, *p* < 0.001], where males consumed less alcohol than females, and of treatment [*F*_(1, 32)_ = 13.51, *p* < 0.001], indicating that both females and males in the synbiotic groups show less preference for alcohol than those in the water groups.

### Effects of Synbiotic Treatment on Behavioral Alterations Following Alcohol Deprivation

**Figure 4:**
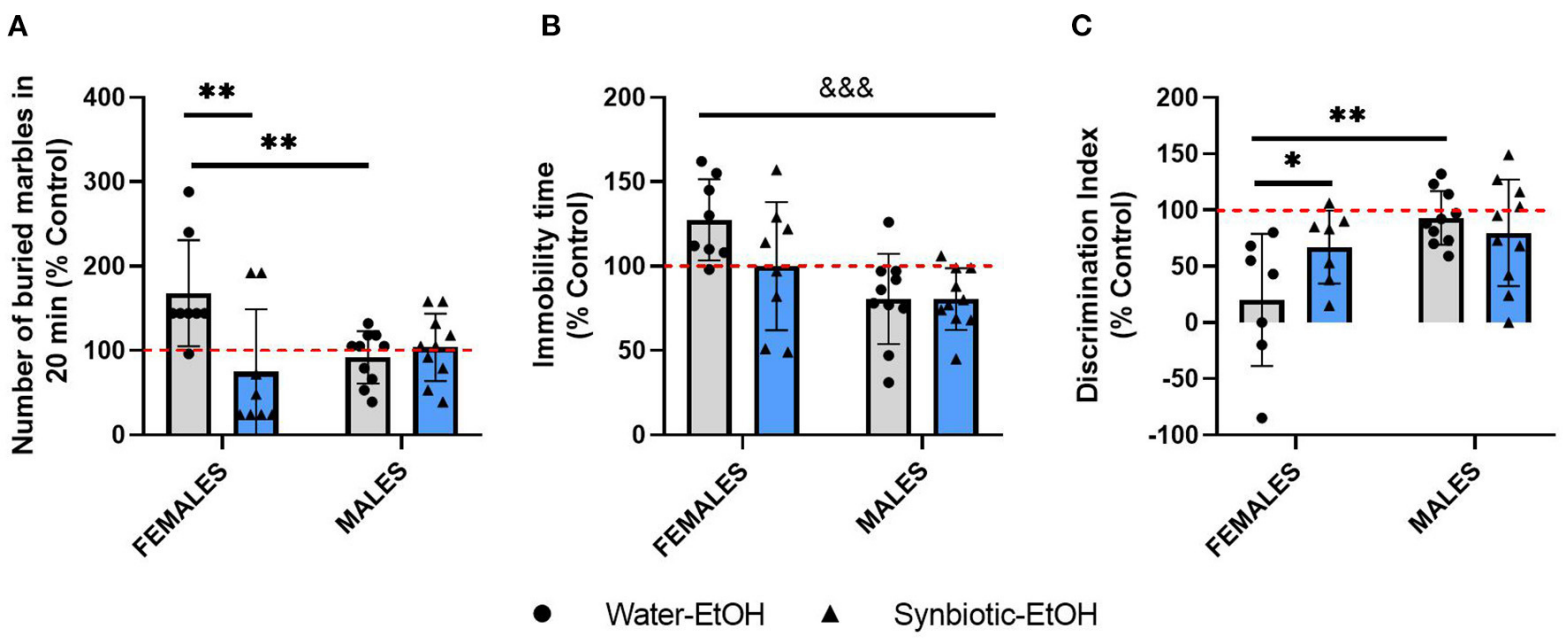
Behavioral alterations following alcohol deprivation in female and male mice exposed to water-alcohol (gray bars and round symbols), or synbiotic-alcohol (blue bars and triangular symbols). **(A)** In the marble burying (MB) test, females in the water-alcohol group showed an anxiogenic-related effect (increased marble-burying) as compared with control mice, and this effect was prevented in the synbiotic-alcohol group (*p* < 0.01). Also, female mice in the water-alcohol group showed more anxiogenic-like behavior than males (*p* < 0.01). **(B)** In the tail suspension (TS) test, females showed more depressive-like behavior (increased immobility) than males (*p* < 0.001). **(C)** In the novel object recognition (NOR) test, females in the water-alcohol group showed deficits in long-term recognition memory (decreased discrimination index scores), but not males (*p* < 0.01), and the deficits observed in females in the water-alcohol group were abolished in the synbiotic-alcohol group (*p* < 0.05). ^&&&^*p* < 0.001 (main effect of sex); *p < 0.05, **p < 0.01 (LSD *post-hoc* test following significant interaction in two-way ANOVA).

After evaluating the effects of alcohol deprivation on relapse to alcohol intake, mice underwent behavioral testing in different paradigms. The data for all the tests from females and males exposed to alcohol-water or alcohol-synbiotic were calculated as % change from the control group (treated only with water throughout the procedure) and were included in the same analyses. In the MB test (Figure 4A), statistical analysis revealed a significant interaction between sex and treatment factors [*F*_(1, 32)_ = 8.77, *p* < 0.01], and a significant treatment effect [*F*_(1, 32)_ = 5.24, *p* < 0.05]. Females in the water-alcohol group displayed more anxiogenic-like behavior (more buried marbles) with respect to the control group, but this effect was significantly lower in the synbiotic-alcohol group (*p* < 0.01). In addition, significantly less anxiogenic behavior was revealed in males in the water-alcohol group with respect to females (*p* < 0.01). In the TS (Figure 4B), a significant effect of sex was revealed [*F*_(1, 32)_ = 13.36, *p* < 0.001], indicating that females showed more depressive-like behavior (increased immobility) than males. In the NOR test (Figure 4C), a significant interaction between factors was observed [*F*_(1, 30)_ = 4.27, *p* < 0.05], and a significant sex effect [*F*_(1, 30)_ = 8.58, *p* < 0.01]. Females in the water-alcohol group showed lower scores in the NOR test vs. the control group, but this effect was not observed in males. In addition, significant differences were observed between females in the water-alcohol group vs. the synbiotic-alcohol group (*p* < 0.05), indicating that synbiotic exposure abolished the longterm recognition memory deficits observed in females during abstinence.

### Effects of Synbiotic Treatment on Gut Microbiota Diversity

**Figure 5:**
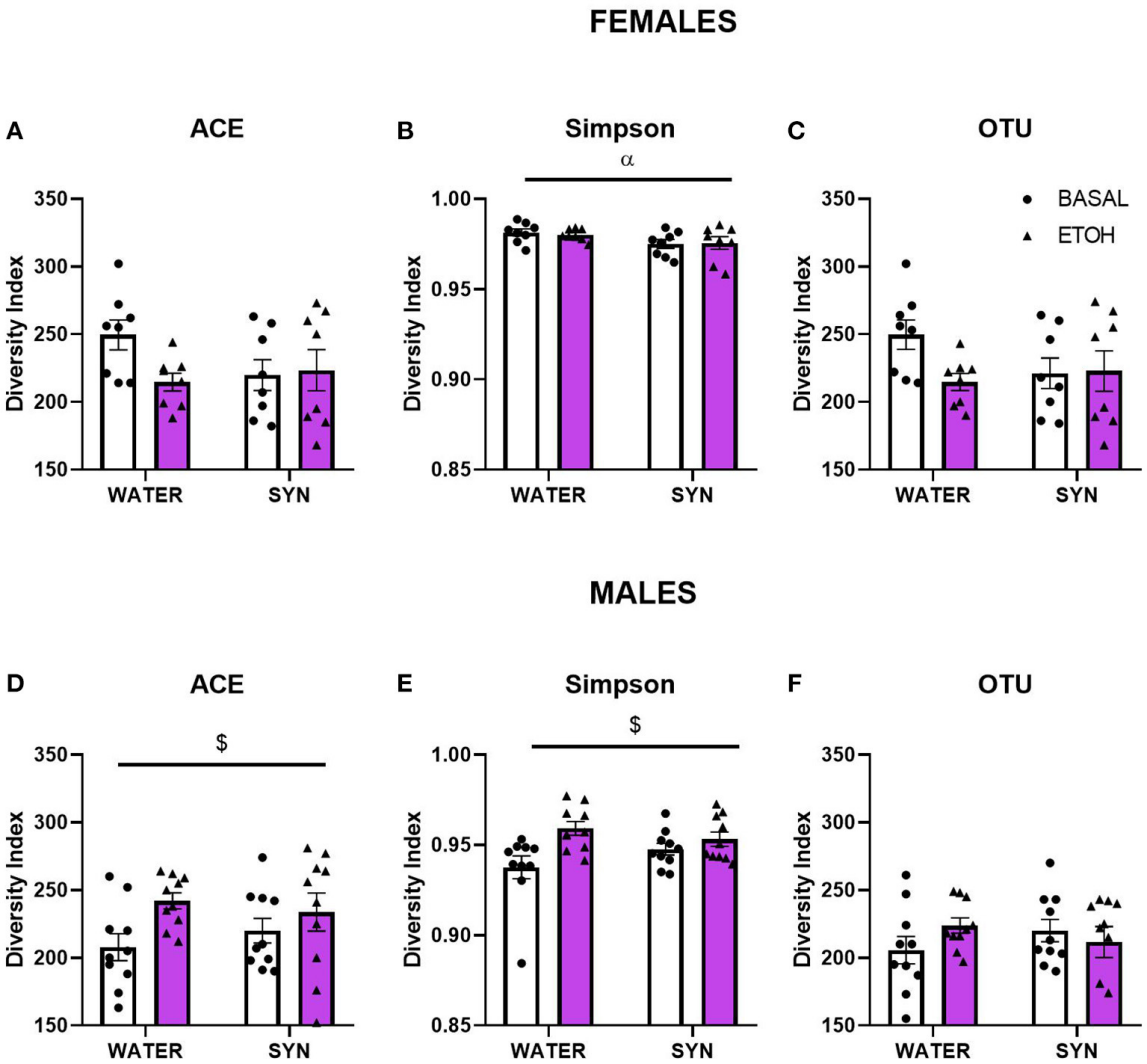
Changes in gut microbiota alpha diversity in female and male mice treated with water or synbiotic (SYN) following 2 weeks of baseline (BASAL; no alcohol; white bars, round symbols), and after 20 days of intermittent access to alcohol (ETOH, violet bars, triangular symbols). **(A,D)** The ACE richness index, **(B,E)** the Simpson diversity index, and **(C,F)** the OTU abundance. ^α^*p* < 0.05 (main effect of treatment); ^$^*p* < 0.05 (main effect of alcohol) following 2-way ANOVA.

**Figure 6:**
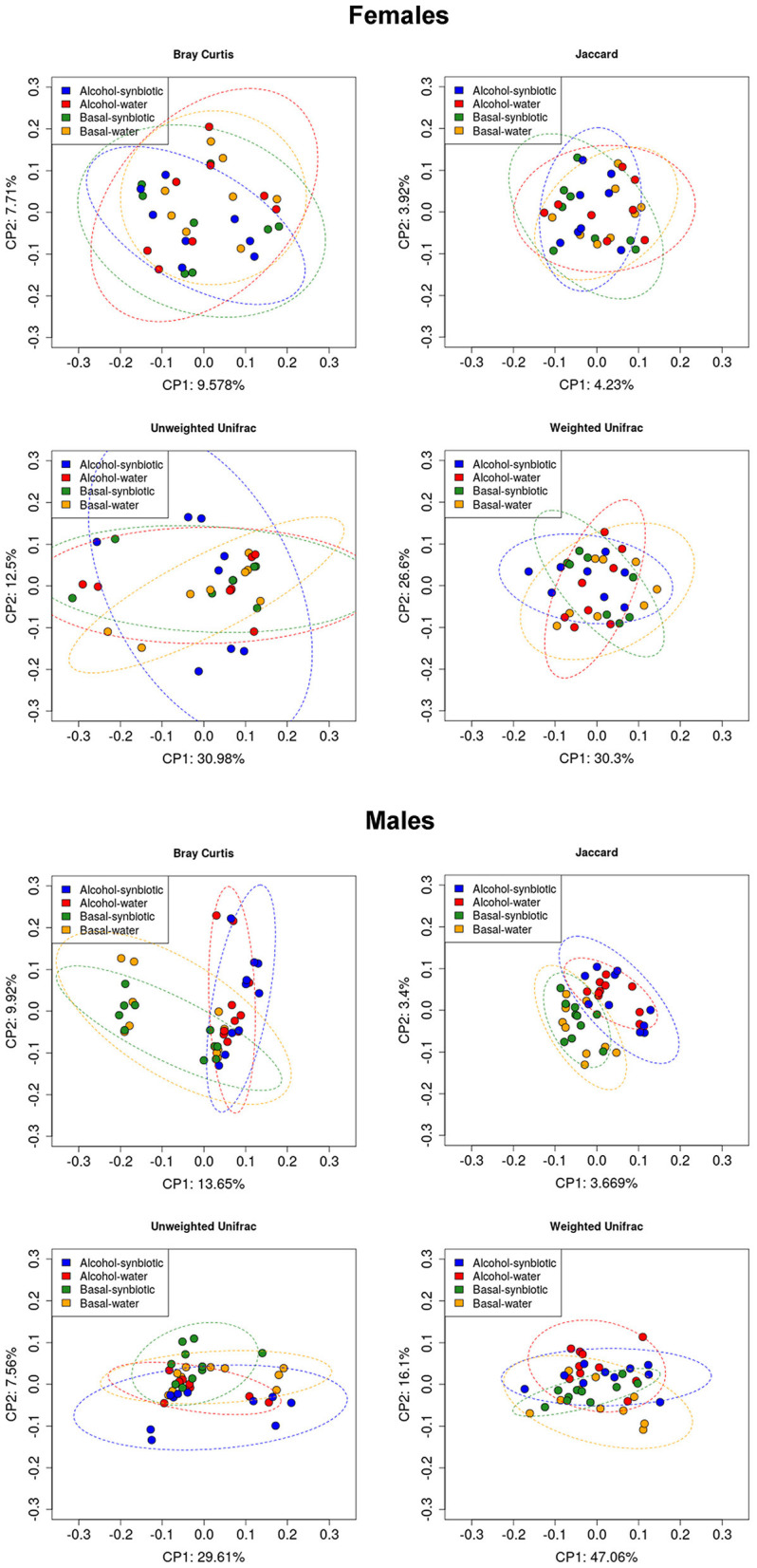
Principal component analysis (PCoA) for microbial community composition and structure (Beta diversity) in fecal samples of females and males under basal conditions (no alcohol), and following intermittent alcohol (15% ethanol solution in water) intake, in mice exposed to water or synbiotic. The data represent Unifrac phylogenetic-based distances for Bray Curtis, Jaccard, Unweighted, and Weighted Unifrac indexes.

To assess whether treatment and behavioral differences were related to changes in the gut microbiota diversity and relative abundance of bacteria present in the fecal samples, we used a 16S metabarcoding approach (see Materials and Methods). In females, the statistical analysis of the alpha diversity index Simpson (Figure 5B), revealed a significant effect of treatment [*F*_(1, 14)_ = 4.85, *p* < 0.05]. No significant differences were observed between groups for the ACE index or OTU abundance in females (Figures 5A,C). On the other hand, in males, the ACE (Figure 5D) and Simpson (Figure 5E) indexes showed a significant alcohol effect [*F*_(1, 18)_ = 6.21, *p* < 0.05; *F*_(1, 18)_ = 7.89, *p* < 0.05, respectively]. No significant effects were observed for OTU abundance in males (Figure 5F). For beta diversity, no significant differences in microbial community composition and structure were observed for the calculated distances between treatments in female mice samples (Figure 6). However, in the case of males, beta diversity analyses based on Unifrac phylogenetic-based distances showed differences in the structure of microbial communities due to the alcohol effect (Figure 6). Differences were observed for Bray Curtis, Jaccard, and unweighted Unifrac distances (Permanova *p* < 0.05), revealing a significant alcohol effect in quality (i.e., presence/absence), and the abundance of phylotypes. No significant differences were detected when using weighted Unifrac distance.

### Effects of Synbiotic Treatment on Gut Microbiota Species Abundance

Under basal conditions in mice exposed only to water, the bacteria that showed a relative abundance >1%, and were more abundant in males than females included: *Akkermansia* uncultured bacterium (females: 4.1 ± 1.95%; males: 14.4 ± 0.87%), *Prevotellaceae UCG-001* (females: 2.32 ± 0.39%; males: 5.09 ± 0.37%), *Alistipes* uncultured bacterium (females: 3.31 ± 0.39%; males: 4.77 ± 0.27%), *Bacteroides* (females: 2.26 ± 0.39%; males: 4.1 ± 0.57%), *Muribaculum* uncultured bacterium (females: 1.03 ± 0.09%; males:1.57 ± 0.14%). In contrast, *Ruminococcaceae* UCG-014 (females: 3.37 ± 0.29%; males: 2.43 ± 0.42%), *Lachnospiraceae* NK4A136 (females: 5.35 ± 0.54%; males: 4.09 ± 0.52%), and *Burkholderiales* bacterium YL45 (females: 3.1 ± 1.06%; males: 4.17 ± 0.45%) showed similar relative abundance in both sexes. Finally, *Lactobacillus* was more abundant in females than males (females: 6.06 ± 0.01%; males: 2.18 ± 0.01).

**Figure 7:**
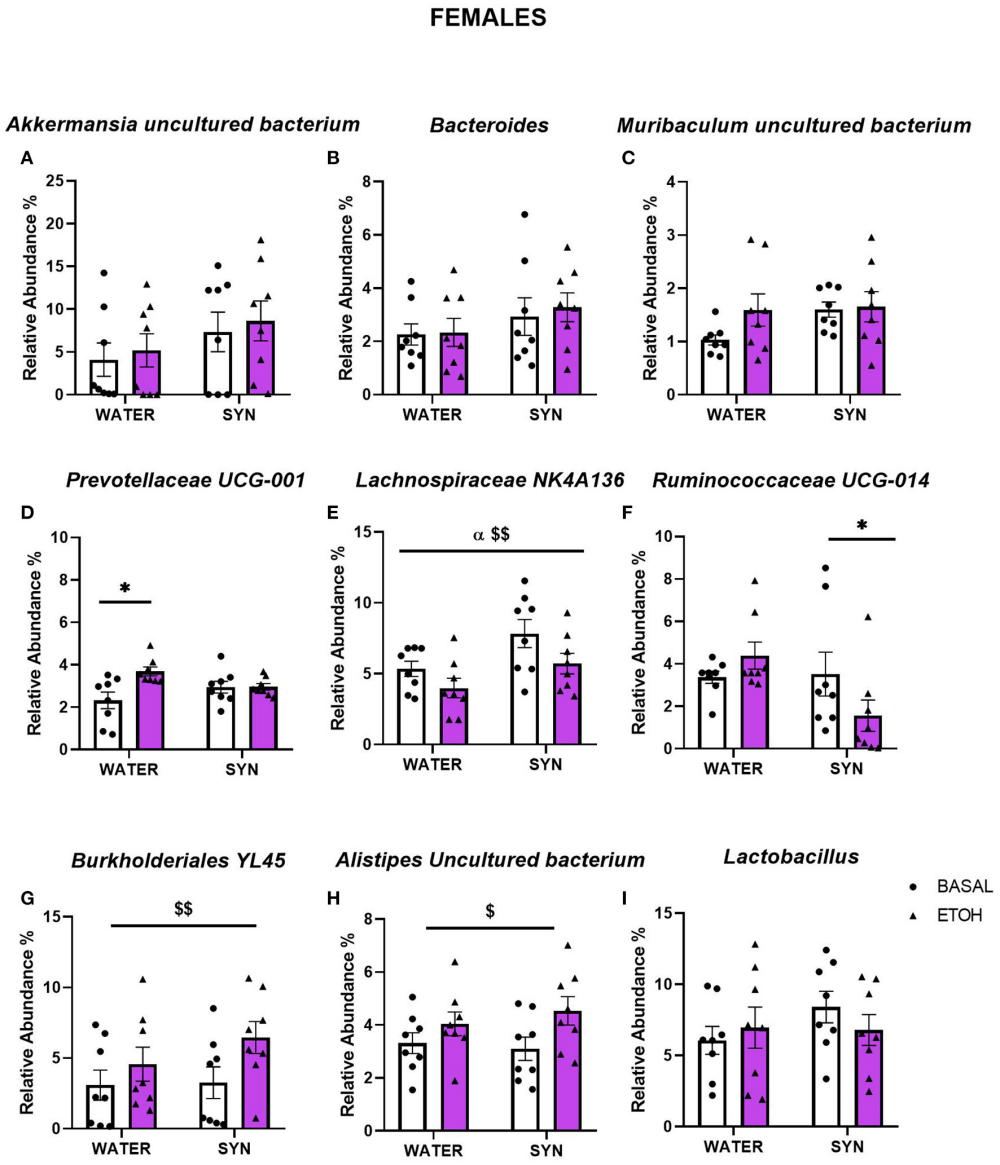
Changes in gut microbial species relative abundance in female mice treated with water or synbiotic (SYN) following 2 weeks of baseline (BASAL; no alcohol; white bars, round symbols), and after 20 days of intermittent access to alcohol (ETOH, violet bars, triangular symbols). No significant changes were observed in *Akkermansia uncultured bacterium* **(A)**, *Bacteroides* **(B)**, or *Muribaculum uncultured bacterium* **(C)**. *Prevotellaceae* UCG-001 abundance was significantly increased by alcohol intake only in the group exposed to water (*p* < 0.05) **(D)**. For *Lachnospiraceae* NK4A136, synbiotic exposure increased abundance under basal conditions, and alcohol decreased relative abundance in both the water and the synbiotic groups **(E)**. *Ruminococcaceae* UCG-014 was significantly decreased only in the synbiotic group (*p* < 0.05) **(F)**. Alcohol significantly increased *Burkholderiales bacterium* YL45 **(G)**, and *Alistipes uncultured bacterium* **(H)** abundance in both the water and synbiotic groups (*p* < 0.01 and *p* < 0.05), respectively. Finally, *Lactobacillus* abundance **(I)** was not significantly modified by alcohol or synbiotic treatment. ^$^*p* < 0.05; ^$$^*p* < 0.01 (main effect of alcohol). ^α^*p* < 0.05 (main effect of treatment). *p < 0.05 (LSD *post-hoc* test following significant interaction in two-way ANOVA).

In females, statistical analyses comparing bacterial abundance in mice exposed to water or synbiotic under basal conditions and following intermittent alcohol consumption revealed no significant changes in *Akkermansia* uncultured bacterium (Figure 7A), *Bacteroides* (Figure 7B), or *Muribaculum* uncultured bacterium (Figure 7C). For *Prevotellaceae* UCG-001, a two-way ANOVA showed a significant effect of alcohol exposure [*F*_(1, 14)_ = 6.86, *p* < 0.05], and a significant interaction between alcohol and treatment [*F*_(1, 14)_ = 6.56, *p* < 0.05]. Alcohol intake significantly increased the abundance of this bacteria in the water group with respect to baseline (*p* < 0.05), but not in the synbiotic group (Figure 7D). For *Lachnospiraceae* NK4A136 (Figure 7E), alcohol [*F*_(1, 14)_ = 8.72, *p* < 0.01] and synbiotic [*F*_(1, 14)_ = 5.61, *p* < 0.05] exposure showed significant effects, but no significant interaction between factors was observed. Synbiotic exposure increased abundance under basal conditions (no alcohol), and alcohol decreased relative abundance in both the water and the synbiotic groups. For *Ruminococcaceae* UCG-014 (Figure 7F), a significant interaction between alcohol and treatment was revealed [*F*_(1, 14)_ = 4.81, *p* < 0.05], and subsequent analysis showed that abundance was significantly decreased only in the synbiotic group (*p* < 0.05). Finally, a significant effect of alcohol was revealed for *Burkholderiales* bacterium YL45, [*F*_(1, 14)_ = 16.52, *p* < 0.01] (Figure 7G), and *Alistipes* uncultured bacterium [*F*_(1, 14)_ = 7.15, *p* < 0.05] (Figure 7H), where alcohol increased their relative abundance in both treatment groups. No significant effects of alcohol or synbiotic exposure were observed for *Lactobacillus* relative abundance (Figure 7I).

**Figure 8:**
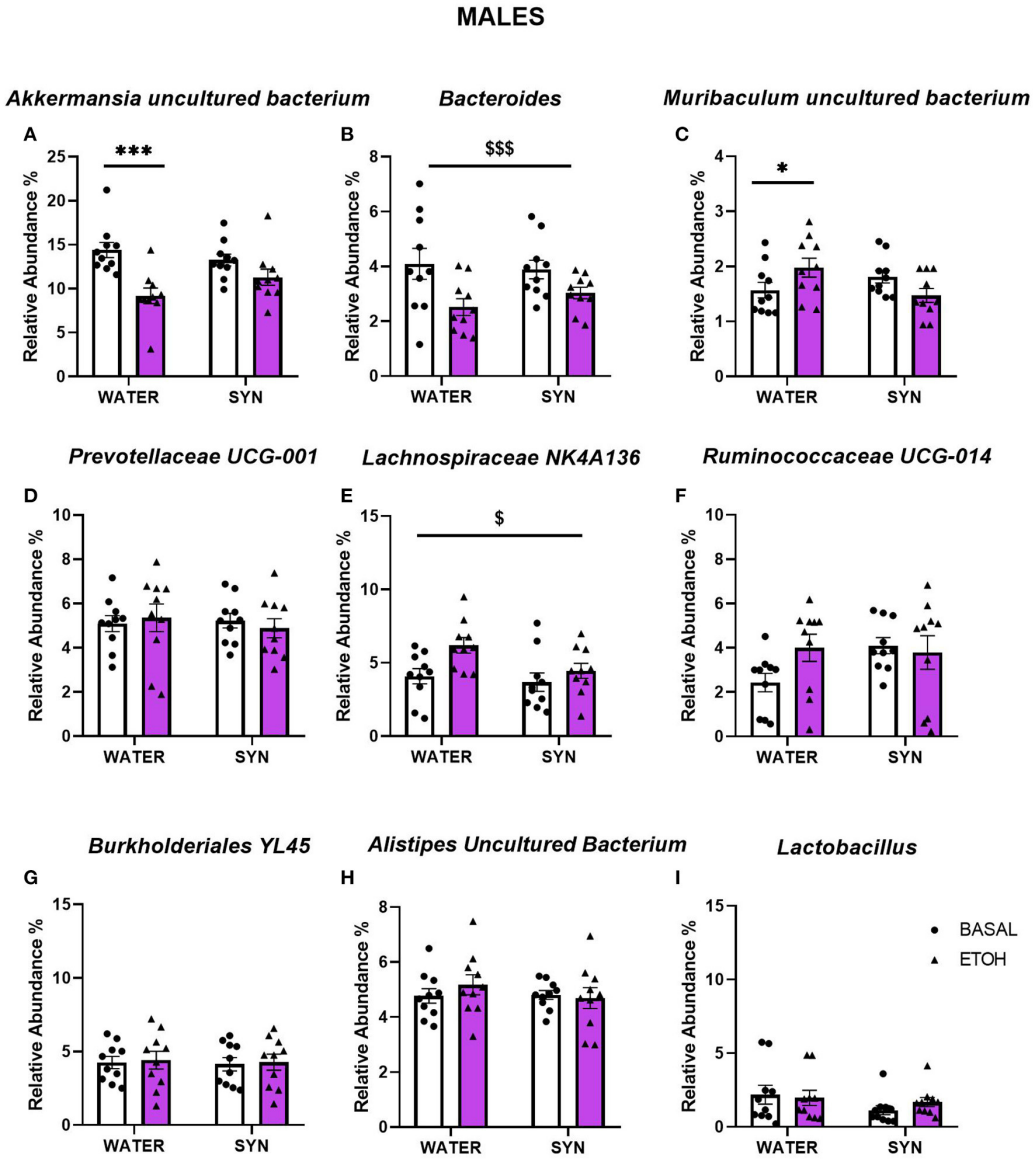
Changes in gut microbial species relative abundance following intermittent alcohol intake in male mice treated with water or synbiotic (SYN) following 2 weeks of baseline (BASAL; no alcohol; white bars, round symbols), and after 20 days of intermittent access to alcohol (ETOH, violet bars, triangular symbols). The abundance of *Akkermansia* uncultured bacterium **(A)** was significantly decreased by alcohol only in the water group (*p* < 0.001). For the *Bacteroides* **(B)**, alcohol-reduced its abundance in both the water and synbiotic groups. Alcohol significantly increased the abundance of *Muribaculum uncultured bacterium* **(C)** only in the water group (*p* < 0.05). Alcohol increased the abundance of *Lachnospiraceae* NK4A136 **(E)** in both the water and the synbiotic groups. No significant changes were observed for *Prevotellaceae* UCG-001 **(D)**, *Ruminococcaceae* UCG-014 **(F)**, *Burkholderiales* bacterium YL45 **(G)**, *Alistipes* uncultured bacterium **(H)**, or *Lactobacillus* **(I)** abundance. ^$^*p* < 0.05; ^$$$^*p* < 0.001 (main effect of alcohol). *p < 0.05, ***p < 0.001(LSD *post-hoc* test following significant interaction in two-way ANOVA).

In males, statistical analyses for *Akkermansia* uncultured bacterium revealed a significant effect of alcohol [*F*_(1, 18)_ = 19.95, *p* < 0.001], and interaction between alcohol and treatment [*F*_(1, 18)_ = 4.13, *p* < 0.05]. Alcohol intake decreased the abundance of this species with respect to baseline conditions in the water group (*p* < 0.001), but not in the synbiotic group (Figure 8A). For the *Bacteroides*, only a significant main effect of alcohol was observed [*F*_(1, 18)_ = 16.85, *p* < 0.001], where lower abundance was observed vs. baseline in both the water and synbiotic groups (Figure 8B). For *Muribaculum* uncultured bacterium (Figure 8C), a significant interaction between alcohol and treatment [*F*_(1, 18)_ = 8.25, *p* < 0.01] was revealed. Alcohol intake significantly increased its abundance in the water group (*p* < 0.05), but not in the synbiotic group. Similarly, for *Lachnospiraceae* NK4A136 (Figure 8E), alcohol increased its abundance mostly in the water group {significant effect of alcohol: [*F*_(1, 18)_ = 6.62, *p* < 0.05]}. For *Prevotella*ceae UCG-001 (Figure 8D), *Ruminococcaceae* UCG-014 (Figure 8F), *Burkholderiales* bacterium YL45, (Figure 8G), *Alistipes* uncultured bacterium (Figure 8H), or *Lactobacillus* (Figure 8I) no significant effects of alcohol or synbiotic exposure were observed.

### Effects of Synbiotic Treatment on SCFA

The concentrations of fecal SCFAs studied in mouse feces before and after chronic alcohol intake are shown in Table 1. Basal levels of butyric acid were higher in females than in males (*p* < 0.001), while propionic acid concentrations were similar in both sexes. Alcohol induced a significant decrease in butyric acid in female mice exposed to water [*F*_(1, 14)_ = 7.64, *p* < 0.05], and this effect was similar in mice exposed to synbiotic. Similarly, propionic acid concentrations were also decreased by alcohol (significant main effect of alcohol [*F*_(1, 14)_ = 9.1, *p* < 0.01], with no significant effect of synbiotic vs. water exposure. In male mice, alcohol and synbiotic exposure significantly increased butyric acid levels (main effect of alcohol: *F*_(1, 18)_ = 7.71, *p* < 0.05; main effect synbiotic: *F*_(1, 18)_ = 8.36, *p* < 0.01). For propionic acid, no significant effects of alcohol, synbiotic, or interaction between these factors were observed in male mice.

**Table 1:** Fecal concentrations of butyric and propionic acids under basal conditions and following alcohol intake in female and male mice exposed to water or synbiotic.

		**Basal**	**Alcohol**
**Females**			
Water	Butyric acid	358.2 ± 43.5	285.8 ± 34.2**^$^**
	Propionic acid	261.3 ± 36.8	195.1 ± 36.8**^$$^**
Synbiotic	Butyric acid	381.2 ± 49.0	287.7 ± 12.0
	Propionic acid	302.0 ± 33.0	227.7 ± 28.4
**Males**			
Water	Butyric acid	164.3 ± 10.0^***^	218.6 ± 14.0**^$^**
	Propionic acid	280.7 ± 21.5	353.5 ± 35.2
Synbiotic	Butyric acid	240.8 ± 30.0	286.3 ± 26.7**^αα^**
	Propionic acid	329.4 ± 26.8	372.3 ± 39.6

$ *p < 0.05*,

$$ *p < 0.01; main effect of alcohol*.

αα *p < 0.01; main effect of treatment (two-way ANOVA)*.

Sex effect,

*** *p < 0.001 (Student T-test). Values are expressed as ng/mg ± SEM*.

### Effects of Synbiotic Treatment on Tryp Metabolites and Neurotransmitter Concentrations in the PFC and HPC

The concentrations of Tryp metabolites and neurotransmitters in the PFC and HPC of females and males following chronic alcohol intake, as well as, the ANOVA F values are shown in Table 2. In the PFC of females, a significant increase in 5-HT concentrations was observed in the water-alcohol vs. control group (*p* < 0.05). 5-HT and Kyn levels also showed significant increases in the synbiotic-alcohol vs. control group (*p* < 0.01 and *p* < 0.05, respectively). In the HPC, Tryp significantly decreased in mice exposed to both water-alcohol (*p* < 0.01) and synbiotic-alcohol (*p* < 0.01) compared with the control group, while GABA significantly decreased only in the water-alcohol vs. the control group (*p* < 0.05). In males, PFC concentrations of NA were decreased in the synbiotic-alcohol group (*p* < 0.05), while GABA levels were decreased in the water-alcohol group (*p* < 0.05) vs. the control group. In the HPC, exposure to water-alcohol induced significant increases in Tryp and Kyn and decreases in GABA levels with respect to the control group (*p* < 0.05 in all cases). On the other hand, 5-HT and NA concentrations in the HPC were reduced in the synbiotic-alcohol group with respect to the control group (*p* < 0.05 and *p* < 0.001, respectively), and to the water-alcohol group (*p* < 0.001).

**Table 2:** Tryptophan pathway metabolites and neurotransmitter concentrations in the prefrontal cortex (PFC) and hippocampus (HPC) of female and male mice following alcohol intake and concomitant exposure to water and synbiotic, and control mice exposed only to water (no alcohol).

		**Control**	**Water-alcohol**	**Synbiotic-alcohol**	***F-*values**
**Females**					
PFC	Tryp	3.67 ± 0.66	5.35 ± 0.57	5.68 ± 0.83	*F*_(2, 17)_ = 1.870, NS
	Kyn	0.06 ± 0.01	0.08 ± 0.01	0.10 ± 0.02^*^	*F*_(2, 16)_ = 3.203, *p* < 0.05
	5-HT	0.43 ± 0.08	0.74 ± 0.08^*^	0.85 ± 0.07^**^	*F*_(2, 17)_ = 7.223, *p* < 0.01
	5-HIAA	2.53 ± 0.24	2.04 ± 0.29	2.23 ± 0.50	*F*_(2, 18)_ = 0.395, NS
	DA	0.12 ± 0.02	0.10 ± 0.01	0.19 ± 0.07	*F*_(2, 17)_ = 2.349, NS
	NA	0.44 ± 0.05	0.41 ± 0.03	0.33 ± 0.03	*F*_(2, 18)_ = 1.871, NS
	GABA	80.98 ± 7.80	65.60 ± 3.89	69.21 ± 6.23	*F*_(2, 19)_ = 1.678, NS
HPC	Tryp	7.82 ± 0.78	5.08 ± 0.55^**^	4.88 ± 0.50^**^	*F*_(2, 19)_ = 6.720, *p* < 0.01
	Kyn	0.06 ± 0.01	0.04 ± 0.01	0.04 ± 0.01	*F*_(2, 19)_ = 2.441, NS
	5-HT	0.56 ± 0.04	0.60 ± 0.04	0.62 ± 0.05	*F*_(2, 18)_ = 0.498, NS
	5-HIAA	3.89 ± 0.17	3.57 ± 0.26	3.31 ± 0.2	*F*_(2, 17)_ = 1.423, NS
	DA	0.06 ± 0.01	0.06 ± 0.01	0.08 ± 0.02	*F*_(2, 16)_ = 0.820, NS
	NA	0.65 ± 0.03	0.76 ± 0.05	0.64 ± 0.04	*F*_(2, 17)_ = 2.287, NS
	GABA	56.66 ± 6.78	42.51 ± 2.97^*^	50.07 ± 2.76	*F*_(2, 17)_ = 2.674, *p* < 0.05
**Males**					
PFC	Tryp	5.26 ± 1.61	7.09 ± 1.15	9.44 ± 1.36	*F*_(2, 20)_ = 2.151, NS
	Kyn	0.10 ± 0.03	0.09 ± 0.01	0.09 ± 0.01	*F*_(2, 21)_ = 0.045, NS
	5-HT	0.82 ± 0.32	0.70 ± 0.12	0.39 ± 0.05	*F*_(2, 21)_ = 2.371, NS
	5-HIAA	2.63 ± 0.28	2.51 ± 0.24	1.84 ± 0.29	*F*_(2, 20)_ = 2.350, NS
	DA	0.10 ± 0.01	1.36 ± 1.01	0.14 ± 0.04	*F*_(2, 21)_ = 0.602, NS
	NA	0.37 ± 0.03	0.32 ± 0.05	0.22 ± 0.03^*^	*F*_(2, 21)_ = 3.572, *p* < 0.05
	GABA	90.29 ± 15.42	57.69 ± 2.77^*^	73.65 ± 8.05	*F*_(2, 22)_ = 3.155, *p* < 0.05
HPC	Tryp	4.64 ± 0.28	5.70 ± 0.25^*^	5.35 ± 0.29	*F*_(2, 21)_ = 3.285, *p* < 0.05
	Kyn	0.017 ± 0.001	0.022 ± 0.001^*^	0.021 ± 0.002	*F*_(2, 19)_ = 3.121, *p* < 0.05
	5-HT	0.50 ± 0.02	0.60 ± 0.09	0.24 ± 0.03^*^^,^ ^ψψψ^	*F*_(2, 22)_ = 9.039, *p* < 0.01
	5-HIAA	3.13 ± 0.28	3.42 ± 0.14	3.25 ± 0.18	*F*_(2, 22)_ = 0.495, NS
	DA	0.05 ± 0.003	0.04 ± 0.01	0.03 ± 0.004	*F*_(2, 21)_ = 1.519, NS
	NA	0.67 ± 0.03	0.57 ± 0.05	0.34 ± 0.03^***^^,^ ^ψψψ^	*F*_(2, 22)_ = 15.33, *p* < 0.001
	GABA	60.06 ± 8.71	40.93 ± 1.10^*^	49.38 ± 5.10	*F*_(2, 23)_ = 3.336, *p* < 0.05

* *p < 0.05;*

** *p < 0.01*,

*** *p < 0.001 vs. control;*

ψψψ *p < 0.001 vs. water-alcohol (LSD post-hoc test following significant one-way ANOVA)*.

*Tryp, Tryptophan; Kyn, kynurenine; 5-HT, serotonin; 5-HIAA, 5-hidroxyindolacetic acid; DA, dopamine; NA, noradrenaline; GABA, γ-aminobutyric acid. Values are expressed as ng/mg ± SEM*.

### Sex-Dependent Correlations Between Behavioral Alterations, Gut Bacterial Abundance, Tryp Metabolites, and Neurotransmitters in the PFC and HPC

**Figure 9:**
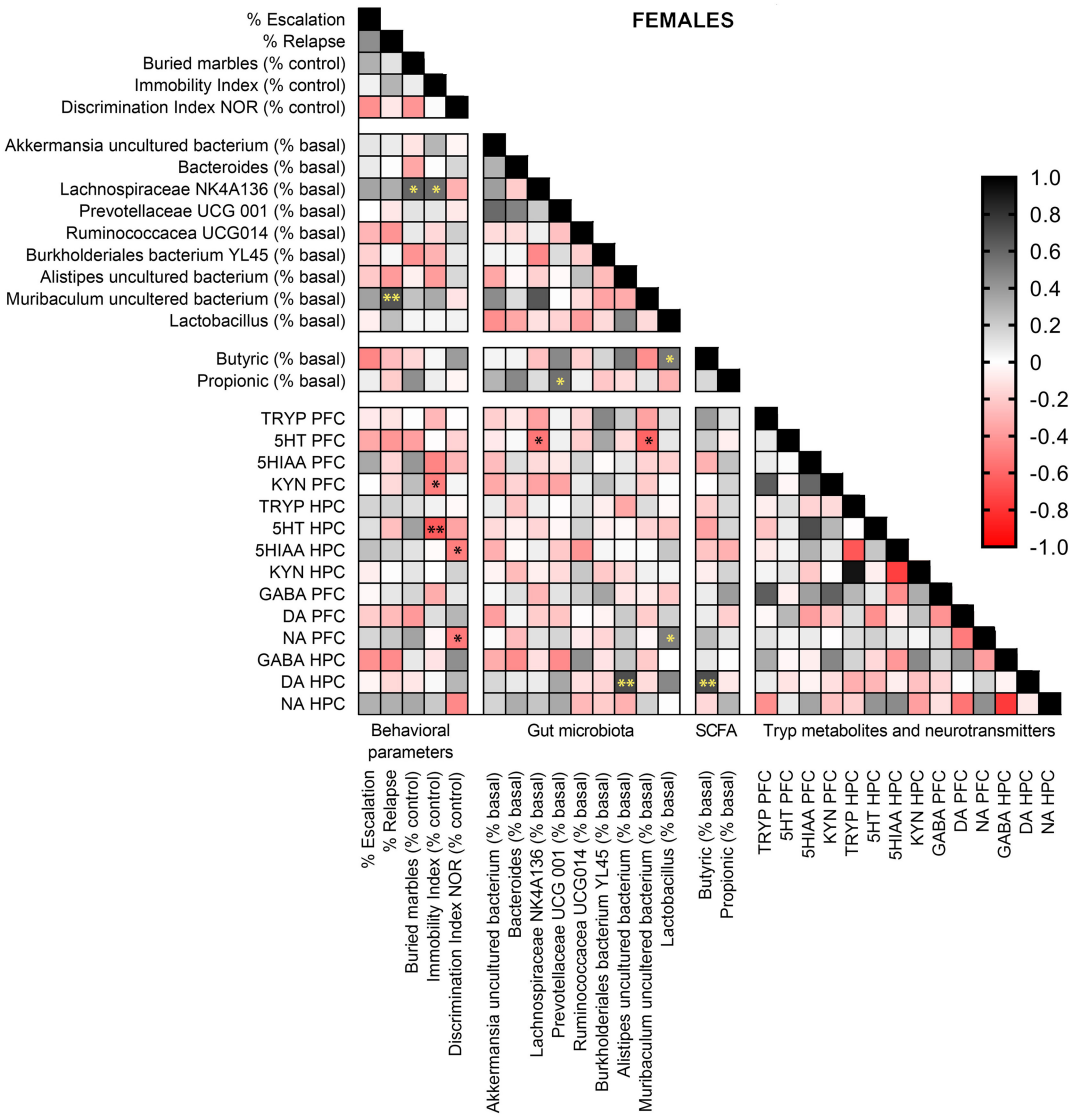
Pearson correlation analysis in female mice exposed to alcohol-water and alcohol-synbiotic comparing behavioral parameters, including escalation (% baseline), relapse (% baseline), depression-like scores (immobility index), anxiety-like scores (buried marbles index), long-term memory (discrimination index in the NOR test) with gut microbial abundance, short-chain fatty acids (SCFA: butyric and propionic acids), tryptophan metabolites: Tryp (tryptophan), serotonin (5-HT), 5-hydroxyindolacetic acid (5-HIAA), kynurenine (KYN), and neurotransmitters including GABA, dopamine (DA) and noradrenaline (NA) concentrations in the prefrontal cortex (PFC) and hippocampus (HPC). Positive correlations are shown in black tones and negative correlations in red tones. Darker tones signify stronger correlations, with significance represented by asterisks (*p < 0.05, **p < 0.01).

Pearson correlation analyses were performed in females and males exposed to alcohol-water and alcohol-synbiotic between behavioral parameters, gut bacterial species abundance, fecal SCFA (butyric and propionic acids), Tryp metabolites, and DA, NA, and GABA concentrations in PCF and HPC. In females (Figure 9), *Muribaculum* uncultured bacterium was positively correlated with relapse to alcohol intake following withdrawal (*r* = 0.665, *p* < 0.01). *Lachnospiraceae* NK 4A136 abundance was positively correlated with anxiety-like behavior (*r* = 0.598, *p* < 0.05) and depression-like scores (*r* = 0.561, *p* < 0.05). In addition, immobility scores were negatively associated with 5-HT concentrations in the HPC (*r* = 0.640, *p* < 0.01), and Kyn levels in the PFC (*r* = 0.503, *p* < 0.05), while memory performance was negatively correlated with NA in the PFC (*r* = 0.524, *p* < 0.05), and 5-HIAA concentrations in the HPC (*r* = 0.505, *p* < 0.05). *Muribaculum* uncultured bacterium (*r* = 0.607, *p* < 0.05), and *Lachnospiraceae* NK 4A136 (*r* = 0.533, *p* < 0.05) abundance were negatively associated with 5-HT levels in the PFC. Also, *Allistipes* uncultured bacteria were positively associated with DA in the HPC (*r* = 0.692, *p* < 0.01), and DA concentrations in the HPC were also positively correlated with fecal levels of butyric acid (*r* = 0.683, *p* < 0.01). Finally, *Lactobacillus* abundance was positively associated with fecal butyric acid levels (*r* = 0.524, *p* < 0.05), and NA concentrations in the PFC (*r* = 0.499, *p* < 0.05), while *Prevotellaceae* UCG 001 abundance was positively correlated with fecal propionic acid concentrations (*r* = 0.554*, p* < 0.05).

**Figure 10:**
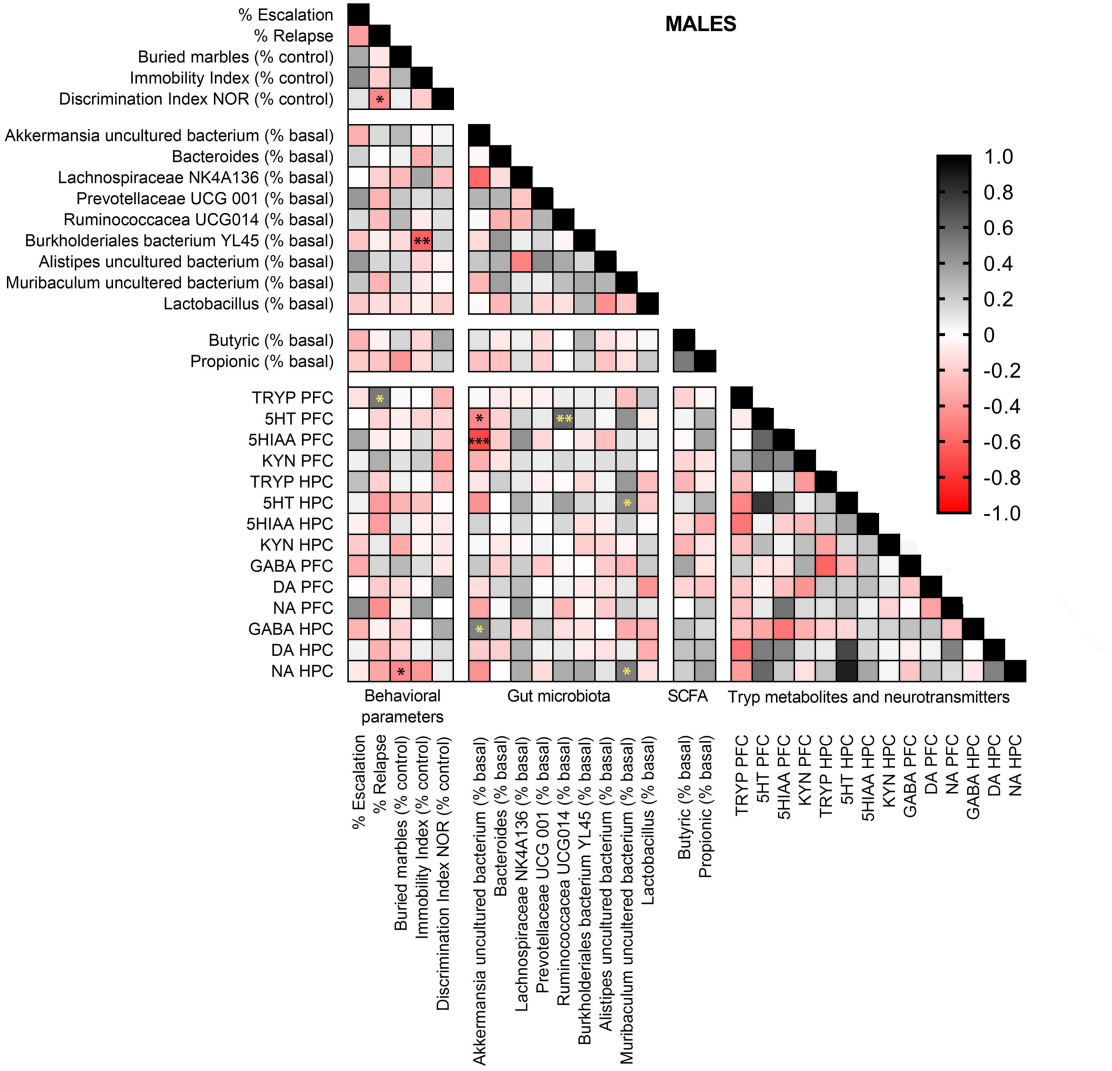
Pearson correlation analysis in male mice exposed to alcohol-water and alcohol-synbiotic comparing behavioral parameters, including escalation (% baseline), relapse (% baseline), depression-like scores (immobility index), anxiety-like scores (buried marbles index), long-term memory (discrimination index in the NOR test) with gut microbial abundance, short-chain fatty acids (SCFA: butyric and propionic acids), tryptophan metabolites: Tryp (tryptophan), serotonin (5-HT), 5-hydroxyindolacetic acid (5-HIAA), kynurenine (KYN), and neurotransmitters including GABA, dopamine (DA) and noradrenaline (NA) concentrations in the prefrontal cortex (PFC) and hippocampus (HPC). Positive correlations are shown in black tones and negative correlations in red tones. Darker tones signify stronger correlations, with significance represented by asterisks (*p < 0.05, **p < 0.01, ***p < 0.001).

In males (Figure 10), Tryp concentrations in the PFC were positively associated with relapse (*r* = 0.524, *p* < 0.05). *Burkholderiales* bacterium YL45 abundance was negatively related with depression-like scores (*r* = 0.616, *p* < 0.01), and anxiety-like scores were negatively associated with NA concentrations in the HPC (*r* = 0.460, *p* < 0.05). *Akkermansia* uncultured bacterium abundance showed negative correlations with 5-HT (*r* = 0.466, *p* < 0.05) and 5-HIAA (*r* = 0.715, *p* < 0.001) levels in the PFC, and was positively correlated with GABA concentrations in the HPC (*r* = 0.499, *p* < 0.05). *Muribaculum* uncultured bacterium abundance was positively associated to 5-HT (*r* = 0.424, *p* < 0.05) and NA (*r* = 0.503, *p* < 0.05) concentrations in the HPC. Finally, *Ruminococcaceae* UCG-014 relative abundance was positively correlated with 5-HT concentrations in the PFC (*r* = 0.581, *p* < 0.01).

## Discussion

In this study, we found that synbiotic dietary supplementation during chronic intermittent alcohol drinking reduced escalation and relapse-like behaviors in mice of both sexes. In parallel, the affective and cognitive alterations that were observed in female mice following alcohol deprivation were abrogated. Overall, these results suggest that modulation of gut microbiota through synbiotic exposure may be beneficial for averting alcohol-related addictive behaviors. In support of the idea that these effects are mediated through the brain-gut-axis, we observed that synbiotic exposure modulated the changes in the relative abundance of gut bacteria, Tryp metabolites, GABA, and NA concentrations in the PFC and HPC occurring following alcohol withdrawal in a sex-specific manner. Moreover, correlation analysis of the pooled data from mice drinking alcohol exposed to water and synbiotic revealed marked sex differences between changes in the relative abundance of specific gut bacteria, brain concentrations of Tryp metabolites, NA and GABA, and behavioral alterations. These data reveal that gut microbiota modulation by synbiotic dietary supplementation may affect differently the gut-brain-axis during chronic alcohol intake in females and males.

As previously reported, we found that chronic intermittent alcohol drinking during 3 weeks induced an escalation of this behavior, and increased the preference ratio for alcohol in female and male mice concomitantly exposed to water (31, 43). This mouse model of access to alcohol is relevant to the human condition, where intermittent consumption of alcohol is regarded as a principal characteristic of human alcohol dependence, and the escalation effect is associated with loss of control over drug-taking found in human alcoholics (44). In line with previous studies in mice using a similar protocol, we observed that female mice exposed to water showed higher basal alcohol drinking (31, 43). Interestingly, exposure to synbiotic reduced the escalation behavior and the increase in preference ratio in both sexes, but this effect was smaller in female mice. In this sense, the larger amounts of alcohol consumed by female mice may have precluded a clear effect of the synbiotic in this population.

During the imposed abstinence phase, mice continued to consume water or synbiotic *ad libitum* for 7 days, and they were then tested for an alcohol deprivation effect by presenting the alcohol solution again to the animals. In this test, an increase in drinking is observed in the first 2–4 h after exposure, which has been associated with relapse-like behavior (31, 45). We found that both males and females showed relapse-like behavior, with females presenting a stronger alcohol deprivation effect than males. Dietary synbiotic supplementation prevented these effects in female mice, while it only diminished the preference ratio in male mice. Our findings are consistent with previous studies showing that females are more susceptible than males to the addictive-like properties of drugs of abuse such as alcohol and cocaine (46, 47), and suggest that synbiotic treatment during alcohol exposure may prevent the loss of control over alcohol taking and craving in females.

Subsequently, we evaluated the carry-over behavioral consequences of escalated alcohol drinking followed by forced abstinence and acute relapse. In female mice, the group exposed to water showed anxiogenic-like behavior, reduced long-term memory performance, and a tendency for a depressive-like effect, and synbiotic treatment reduced these alterations. In males, however, no significant affective or cognitive alterations were observed following withdrawal, suggesting that male mice may need a higher intake of alcohol to observe these effects. Although there is some evidence pointing to the beneficial effects of prebiotics, probiotics, or their combination (synbiotics) on alcohol-related alterations in intestinal permeability (48, 49), chronic psychosocial stress (50), and obesity (51), fewer studies are available supporting the potential benefits of these dietary supplements for the affective and cognitive deficits related with alcohol withdrawal. However, there is accumulating evidence showing that induced gut microbiota dysbiosis with antibiotics or fecal transplantation affects behavioral responses to cocaine and chronic alcohol intake (24, 25, 52).

It has been extensively described that drugs of abuse, including alcohol, cocaine, and methamphetamine produce an imbalance of gut microbiota in humans (19–21, 53), and animal models (25, 48, 54–57). Although sex differences in addiction-related behaviors and associations with gut bacterial abundance have been recently reported (58), there is a lack of studies investigating sex differences in the effects of alcohol on gut bacteria composition. In our study, we found that chronic intermittent alcohol drinking in male mice increased the richness of microbiota (i.e., increased ACE index) in a similar manner in the group exposed to water and synbiotic, while it decreased its diversity (i.e., increased Simpson index). Similarly, beta diversity analysis showed that both groups exposed to alcohol clustered separately with respect to the basal conditions (no alcohol present). In agreement, previous data show that chronic alcohol exposure in male mice modulates the beta diversity of intestinal microbiota and the bacterial profile (25). In females, however, beta diversity was highly variable, with no alcohol effect, while alpha diversity analysis showed that synbiotic exposure increased gut microbiota diversity (i.e., decreased Simpson index) in both basal conditions (no alcohol) and following chronic alcohol drinking.

Moreover, there is evidence suggesting that the relative abundance profile of gut bacteria differs in males and females (59). In accordance, we found that male mice show higher levels of fecal *Akkermansia, Bacteroides, Muribaculum, Prevotella*, and *Alistipes*, and lower levels of *Ruminococcaceae* UCG-014 than females, while the relative abundance of *Lachnospiraceae* NK4A136 and *Burkholderiales* YL45 were similar in both sexes. Notably, the relative abundance of certain types of bacteria was modulated by alcohol in a sex-dependent manner. In male mice, alcohol decreased *Akkermansia* uncultured bacterium, and *Bacteroides*, while it increased the abundance of *Muribaculum* uncultured bacterium. Other research has associated decreases in the abundance of *Akkermansia* muciniphila and *Bacteroides* mostly with obesity in humans (60–62), and laboratory animals (62). Furthermore, in male mice, an acute challenge with a high dose of alcohol (30% w/v, 6 g/kg body weight), reduces *Akkermansia muciniphila* bacteria (63). Finally, higher levels of certain types of *Muribaculum* bacteria have been found to predict recognition memory impairments in female rats exposed to intermittent cafeteria diet (64). Alternatively, in females, alcohol significantly increased the abundance of *Ruminococcaceae* UCG-001, *Ruminococcaceae* UCG-014, *Burkholderiales bacterium YL45*, and *Alistipes*, but decreased the abundance of *Lachnospiraceae* NK 4A136. Importantly, in males, synbiotic exposure specifically restored the changes in abundance induced by alcohol intake in *Akkermansia* and *Muribaculum*, while in females it affected more profoundly the changes induced in *Prevotella*ceae UCG-001 and *Ruminococcaceae* UCG-014. Although it has been well documented that alcohol administration induces gut dysbiosis in animals and humans (18–22), to our knowledge, this is the first study showing sex-dependent alterations in specific gut bacterial abundance by alcohol intake in mice. Interestingly, synbiotic treatment induced sex-dependent effects in terms of bacterial abundance mostly following chronic intermittent alcohol intake, and not during basal conditions. Previous preclinical and human studies have documented sex differences both in the baseline profile of gut microbiota, and in the effects of diet and/or probiotic administration in terms of the composition of bacteria and its metabolic products, providing evidence for genotype by diet interactions (65, 66). Our data provide evidence for a triple genotype-alcohol-synbiotic interaction on gut microbiota profile. The responses observed in our study could be attributed to differences in hormone levels or in the immune system between sexes, although further investigations are necessary to fully understand the mechanisms involved in these associations.

Gut microbiota produces SCFAs (propionate, and butyrate) as metabolic end products of dietary fiber and carbohydrate fermentation (67). Currently, an increasing amount of evidence points to the potential connection between diet, microbiota diversity, and SCFAs in health and disease [see (68) for review]. In this sense, butyric acid production by gut microbiota has been suggested to ameliorate intestinal inflammatory processes (69), and may have beneficial effects in depressive states (70). In addition, chronic ethanol consumption in mice results in changes in microbiota diversity and alters SCFA production (71). Another study shows that concomitant exposure of alcohol and fermented rice liqueur containing yeast and lactic acid bacteria, increased fecal SCFAs (72). In this study, alcohol consumption in females resulted in decreased fecal concentrations of butyric and propionic acids in both water and synbiotic-exposed mice. In contrast, alcohol increased fecal concentrations of both SCFAs in males, and synbiotic intake did not significantly modify these effects. These findings suggest that higher doses of synbiotic supplementation may be needed to observe relevant effects on fecal SCFAs.

Tryptophan is an essential amino acid, the precursor of 5-HT and Kyn (73). Most of the ingested Tryp is absorbed in the small intestine but some of it can reach the large intestine, where it is metabolized by gut microbiota. Thus, it has been reported that commensal bacteria have a pivotal role in the availability of intestinal Tryp, and as regulators of its metabolism [reviewed in (74)]. In addition, Tryp and Kyn can cross the brain-blood barrier acting as neuromodulators and have been implicated in several neuropsychiatric diseases (75). We investigated whether changes in gut bacteria composition by chronic alcohol intake and/or synbiotic supplementation would alter Tryp metabolism and neurotransmitter concentrations in the PFC and the HPC of female and male mice. In females, we observed that both alcohol and synbiotic treatment decreased Tryp levels in the HPC and increased 5-HT concentrations in the PFC. In males, Tryp and Kyn levels in the HPC were increased by alcohol, but not by synbiotic treatment, while 5-HT production was decreased by synbiotic and not by alcohol. These data are consistent with other studies showing that modulation of gut microbiota can lead to changes in 5-HT and Kyn metabolism in the CNS (13, 76), and sex-dependent alterations in the serotonergic system have been reported in the HPC of mice lacking intestinal microbiota (germ-free mice) (77).

In this study, intermittent access to alcohol exerted sex-dependent changes in NA and GABA concentrations in the PCF and HPC, and differential regulation of these effects by synbiotic treatment in both sexes. GABA levels were significantly reduced by alcohol only in the HPC of females, while in males, reductions were observed in both the PFC and HPC. On the other hand, NA levels were significantly reduced by synbiotic treatment in the PFC and HPC of males only. Chronic administration of alcohol has been shown to induce dysregulations in central 5-HT, NA, and GABA neurotransmission. Hence, in pre-clinical studies acute alcohol administration results in an increase of 5-HT levels in various brain structures involved in reward processes, and NA metabolites in the brain are increased following acute alcohol administration in humans, which remain high even after withdrawal [see (78) for review]. In addition, an extensive amount of studies have linked NA to the mechanisms of stress-related alcohol effects, and the motivational properties of alcohol [see (78) for review]. Moreover, studies show that the administration of the probiotic *Lactobacillus rhamnosus* JB-1 increases brain GABA levels in mice (79), and alters the expression of GABA receptors in the amygdala and HPC (30).

Several significant sex-dependent associations were found between alterations in behavioral parameters, changes in bacterial abundance, and brain Tryp metabolites and neurotransmitter concentrations in mice exposed to alcohol-water and alcohol synbiotic. Thus, in females, the percent increase in relapse with respect to controls positively correlated with changes in *Muribaculum* uncultured bacterium abundance, where most mice in the synbiotic group showed less relapse when the abundance of this bacterial species was lowest. Also, *Lachnospiraceae* NK 4A136 abundance was associated with more anxiety and depression-like behaviors. Moreover, *Muribaculum* uncultured bacterium and *Lachnospiraceae* NK4A136 abundance predicted lower concentrations of 5-HT in the PFC of females. In line with these data, previous reports have linked the *Lachnospiraceae* bacterial family with anxiety, depression, and stress exposure (80–83). In addition, lower concentrations of 5-HT in the HPC, and Kyn in the PFC were associated with depression states, while lower NA and 5-HIAA production in the HPC predicted memory impairments. In males, less abundance of *Burkholderiales* bacterium YL45 was associated with more depression-like responses. In addition, higher PFC Tryp levels were associated with more relapse, and lower NA levels in the HPC predicted less anxiety-like responses. Also in males, *Akkermansia* uncultured bacterium and *Ruminococcaceae* UCG014 abundance were associated with changes in 5-HT levels in the PFC, while *Akkermansia* and *Muribaculum* uncultured bacteria were positively correlated with 5-HT, NA, and GABA concentrations in the HPC. Together, these findings suggest a link between changes in bacterial abundance resulting from alcohol intake and central 5-HT, NA, and GABAergic neurotransmission that is modulated by dietary supplementation with a synbiotic mixture, which may regulate the addictive-like behavioral alterations induced by alcohol.

In summary, this work revealed that synbiotic exposure modulated alcohol intake and addiction-related behaviors in female and male mice, and induced sex-dependent changes in bacterial abundance, Tryp metabolism, and neurotransmitter concentrations in the PFC and HPC. These results highlight the need to further investigate the sex-dependent effects of dietary supplementations on alcohol-induced gut microbiota alterations and their impact on addictive behavior. Several limitations of the study should be discussed. The first has to do with the reduced number of subjects used that may hinder the generalization of the results. However, overall the data showed moderate variability, and most statistical analyses revealed significant differences between treatment groups, validating the relevance of the findings. Nonetheless, further studies with more subjects should be necessary to confirm these results. Another limiting factor is the fact that only those bacterial species with more than 1% relative abundance were included in the statistical analyses, thus, important effects may have been missed in those bacteria with low abundance. Furthermore, changes in the functionality of bacteria should be addressed in future studies. In addition, under our experimental conditions, males consumed lower amounts of alcohol than females, which may have indirectly contributed to the lack of post-relapse behavioral alterations observed in males. However, alcohol intake in males did modulate gut bacterial abundance and brain neurotransmitter levels, and significant associations between these parameters were observed.

## Data Availability Statement

The datasets presented in this study can be found in online repositories. The names of the repository/repositories and accession number(s) can be found below: NCBI SRA BioProject, accession no: PRJNA761096.

## Ethics Statement

The animal study was reviewed and approved by Local Ethical Committee (CEEA-PRBB) and followed the mandatory ethical guidelines (National Institutes of Health 1995; European Communities Directive 86/609 EEC).

## Author Contributions

PR and RT: funding acquisition. PR, EK, and NP: experimental design. EK, EV, and CF: animal experiment, behavioral procedures, and sample collection. PG and TG: metagenomics analysis. AG and NP: metabolomics analysis. PR, EK, and NP: statistical analyses. PR, RT, and NP: manuscript redaction. All authors contributed substantially to the work and approved the final version of the manuscript.

## Funding

This work was supported by grants from Ministerio de Sanidad, Consumo y Bienestar Social; Delegación del Gobierno para el Plan Nacional Sobre Drogas #2018I026 (PR), and a grant from DIUE de la Generalitat de Catalunya 2017 SGR 138 (RT) from the Departament d'Economia i Coneixement de la Generalitat de Catalunya (Spain).

## Conflict of Interest

PG was employed by company Microomics Systems S.L. The remaining authors declare that the research was conducted in the absence of any commercial or financial relationships that could be construed as a potential conflict of interest.

## Publisher's Note

All claims expressed in this article are solely those of the authors and do not necessarily represent those of their affiliated organizations, or those of the publisher, the editors and the reviewers. Any product that may be evaluated in this article, or claim that may be made by its manufacturer, is not guaranteed or endorsed by the publisher.
