# Supplementary material for: Sex-Specific Effects of Synbiotic Exposure in Mice on Addictive-Like Behavioral Alterations Induced by Chronic Alcohol Intake Are Associated With Changes in Specific Gut Bacterial Taxa and Brain Tryptophan Metabolism
Source: Front Nutr. 2021 Nov 26;8:750333. doi: 10.3389/fnut.2021.750333 (PMC8662823; doi:10.3389/fnut.2021.750333)
Supplement: Supplementary file 1 [file Data_Sheet_1.docx]

Supplementary Material

## Supplementary Figures


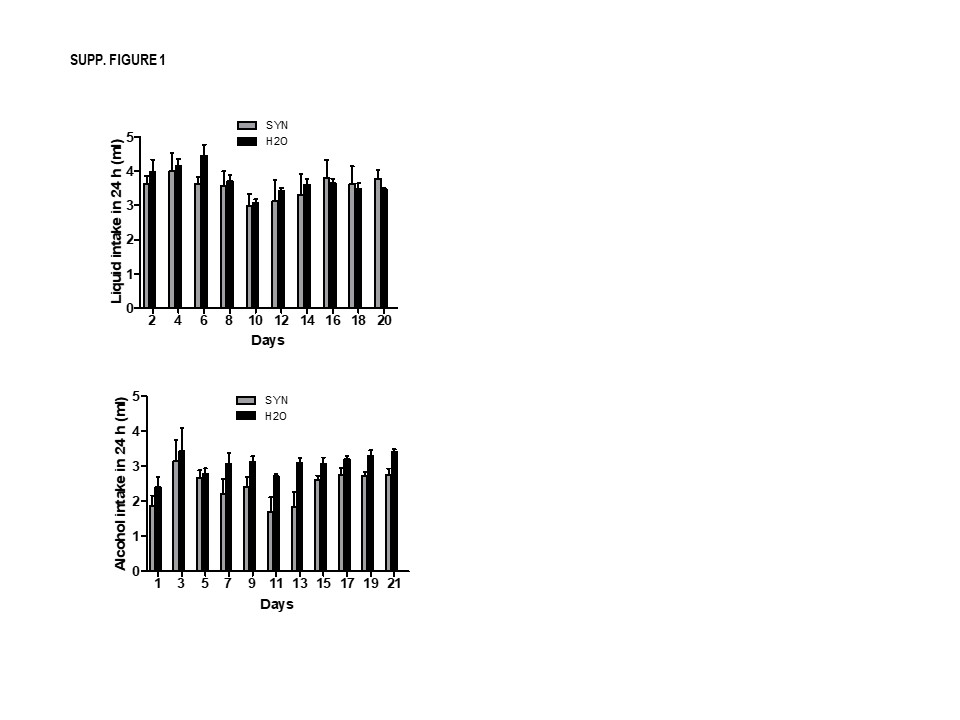


**Supplementary Figure 1.** Pilot study showing the quantities of alcohol (15% ethanol solution in water), water and synbiotic consumed during 21 days. On odd days, group 1 had a choice between two bottles containing either alcohol or water, and group 2 had a choice between two bottles containing either alcohol or a synbiotic solution during 24 h. On even days, group 1 was presented with two bottles containing water, and group 2 with two bottles containing a synbiotic solution.
